# Explicit and Implicit Emotion Processing in the Cerebellum: A Meta-analysis and Systematic Review

**DOI:** 10.1007/s12311-022-01459-4

**Published:** 2022-08-23

**Authors:** Jordan E. Pierce, Marine Thomasson, Philippe Voruz, Garance Selosse, Julie Péron

**Affiliations:** 1 Cognitive and Affective Neuroscience Laboratory, Center for Brain, Biology, and Behavior, University of Nebraska-Lincoln, Lincoln, NE USA; 2 Clinical and Experimental Neuropsychology Laboratory, Department of Psychology, University of Geneva, 40 bd du Pont d’Arve, 1205 Geneva, Switzerland; 3 Neuropsychology Unit, Neurology Department, University Hospitals of Geneva, Geneva, Switzerland

**Keywords:** Cerebellum, Emotion, Meta-analysis, fMRI, Explicit attention, Implicit attention

## Abstract

The cerebellum’s role in affective processing is increasingly recognized in the literature, but remains poorly understood, despite abundant clinical evidence for affective disruptions following cerebellar damage. To improve the characterization of emotion processing and investigate how attention allocation impacts this processing, we conducted a meta-analysis on task activation foci using GingerALE software. Eighty human neuroimaging studies of emotion including 2761 participants identified through Web of Science and ProQuest databases were analyzed collectively and then divided into two categories based on the focus of attention during the task: explicit or implicit emotion processing. The results examining the explicit emotion tasks identified clusters within the posterior cerebellar hemispheres (bilateral lobule VI/Crus I/II), the vermis, and left lobule V/VI that were likely to be activated across studies, while implicit tasks activated clusters including bilateral lobules VI/Crus I/II, right Crus II/lobule VIII, anterior lobule VI, and lobules I-IV/V. A direct comparison between these categories revealed five overlapping clusters in right lobules VI/Crus I/Crus II and left lobules V/VI/Crus I of the cerebellum common to both the explicit and implicit task contrasts. There were also three clusters activated significantly more for explicit emotion tasks compared to implicit tasks (right lobule VI, left lobule VI/vermis), and one cluster activated more for implicit than explicit tasks (left lobule VI). These findings support previous studies indicating affective processing activates both the lateral hemispheric lobules and the vermis of the cerebellum. The common and distinct activation of posterior cerebellar regions by tasks with explicit and implicit attention demonstrates the supportive role of this structure in recognizing, appraising, and reacting to emotional stimuli.

**Supplementary Information:**

The online version contains supplementary material available at 10.1007/s12311-022-01459-4.

## Introduction

The cerebellum has long been associated with motor domain functions such as gait control and movement adaptation. In recent years, however, studies increasingly have identified functions of the cerebellum that extend beyond the motor domain and encompass a multitude of cognitive functions, as well as, notably, affective processes [10, 56, 65, 66, 72, 85]. Furthermore, several anatomical and functional connectivity studies have demonstrated that the cerebellum is reciprocally connected with diverse cortical and subcortical regions subserving multiple functional domains [6, 11, 28, 29, 44, 46, 50, 73], allowing the cerebellum to influence affective processes such as emotion appraisal by modifying activity in the relevant pathways. To understand these affective brain networks and the dynamic feedforward/feedback processes that generate and recognize emotions, it is therefore critical to understand the contribution and organization of the cerebellum, which ultimately can lead to improved clinical approaches to cerebellar disease or injury.

### More Than Motor Control

Current theories propose that the cerebellum produces a modulatory signal that arises from the construction of an internal model of the outcome of actions or thoughts based on the individual’s current state [32, 45, 54, 57, 86]. Comparing the actual outcome to the predicted outcome in a given context allows the cerebellum to provide feedback to the cortex for behavioral optimization. Another, not mutually exclusive, theory for the cerebellum’s function emphasizes the temporal coordination of events, wherein it precisely learns and sequences inputs and outputs to perform well-tuned responses [12, 33, 34], including social sequences where one must mentalize about another’s beliefs to correctly predict their next action [39, 43], such as playing sports or searching for a lost item. While these theories were developed primarily in relation to motor function, the largely uniform neural architecture of the cerebellar hemispheres suggests that the cerebellum performs a comparable function across domains [64, 65], including emotion processing. Alternately, it has been proposed that the cerebellum need not perform only a single type of computation, but rather that by utilizing unique neural algorithms [19] it can accomplish multiple functions across diverse tasks.

### Clinical Evidence of Emotion Processing in the Cerebellum

Early evidence for the role of the cerebellum in emotion processing arose from clinical observations that when the structure is damaged by disease or injury, affective symptoms occur. Initial case studies of dysfunction impacting non-motor cerebellar regions lead to recognition of a “dysmetria of thought” and the cerebellar cognitive affective syndrome [3, 63, 66, 67]. Clinical manifestations of this syndrome include cognitive disruptions and personality changes such as increased impulsivity and aggression, inappropriate laughter, or affective blunting [67, 75]. Furthermore, the cerebellum is often affected in psychological disorders such as autism spectrum disorders, schizophrenia, and depression, possibly in relation to its role in social cognition and mentalizing [9, 14, 40, 42, 79, 81, 82]. For example, in patients with depression or bipolar disorder, molecular changes to Purkinje cells and a reduction in cerebellar volume have been associated with clinical diagnosis, and in healthy individuals have been correlated with symptoms of neuroticism [1, 47, 68].

Clinical findings further suggest that the location of the cerebellar lesion might explain some differences in the observed affective symptoms, according to the type of response or cognitive labelling of the emotion that is required [13]. According to the component process theory of emotion, there are multiple facets of an emotion experience, including physiological responses, action tendencies, and subjective feelings, that interact during the emergence of an emotion and draw upon distributed neural resources depending on current contextual demands [24, 60, 61]. Many tasks require explicit, conscious, direct processing of the emotional content of the stimulus, while others engage implicit, unconscious processing of emotion by directing attention to a non-affective feature or task. Explicit emotion processing requires attention to emotional features and greater cognitive elaboration to recognize and label an emotion, whereas implicit emotion processing involves changes to autonomic responses that can change physiological arousal without awareness – functions that recruit different brain regions [16, 23, 52, 62], including within the cerebellum [30, 62]. Specifically, it has been proposed that explicit attentional processing of emotions may recruit the posterior lateral hemispheres of the cerebellum and implicit processing may recruit the vermis [13]. This proposal is further supported by cerebellar patterns of connectivity with frontal-parietal cortex and the amygdala/brainstem for explicit and implicit emotion processing, respectively [13, 27, 58, 70]. Nonetheless, this functional dichotomy has not been confirmed across affective neuroimaging studies of healthy participants.

### Neuroimaging Meta-analyses of the Emotional Cerebellum

Many previous neuroimaging studies of emotion in healthy adults have been conducted using functional magnetic resonance imaging (fMRI) and positron emission tomography (PET). Yet most studies focused on the role of the amygdala or prefrontal cortex in generating or regulating emotional responses [17, 20, 41, 53, 59, 84]. While these regions undoubtedly are crucial to emotion processing, this emphasis discounts other structures such as the cerebellum or basal ganglia that also modulate activity within the larger affective network [4, 7, 56, 65, 76]. Unfortunately, the cerebellum often is excluded partially or completely from imaging studies in favor of the neocortex, due to technical limitations on the field of view. Even when activations are reported in the cerebellum, these frequently are dismissed as motor-related and not discussed in relation to the affective function assessed by the task.

Several studies have investigated the diverse functional roles of the cerebellum in multiple domains, including a study [26] that looked at motor and non-motor fMRI tasks and resting-state activity in a large sample from the Human Connectome Project. The authors found that the cerebellum contains multiple representations of not only the motor domain, but also cognitive, social, and emotional domains in Crus I and II, and lobules IX/X [26]. This report extended previous findings showing multiple motor maps in the anterior human cerebellum [11, 25] to non-motor functional domains. Moreover, another recent study [36] examined a range of functional tasks in a single sample across multiple fMRI sessions and similarly found several subregions of the cerebellum that responded to a given functional domain, extending across anatomical lobule boundaries.

While such studies of large samples or multiple time points offer detailed information about a few tasks or individuals, a meta-analysis of existing studies of emotion offers a complementary means of building upon and synthesizing findings from multiple tasks and diverse participants to identify common foci of activation supporting a particular functional domain. Previous meta-analyses have investigated non-motor activations within the cerebellum [35, 38, 71, 80] and reported activity in the posterior cerebellum for several functions, including emotion, social cognition, language, timing, and working memory, that was distinct from anterior lobe representations of sensorimotor functions. Specifically, Keren-Happuch et al. [35] reported that emotion was associated most consistently with activity in bilateral lobule VI and Crus I. Van Overwalle and colleagues [74] focused on the cerebellum’s role in social cognition (a construct that often overlaps or relies upon affective processing), including mirroring behavior and theory of mind, and reported greater involvement when tasks required abstract mentalizing about another person’s traits or social category as well as past or future events (see also [81, 82]). Most recently, Klaus and Schutter [37] conducted a meta-analysis investigating anger and aggressive behavior and reported related activation peaks within the bilateral posterior lobules and anterior somatomotor regions of the cerebellum, respectively.

### The Current Meta-analysis

Building on the existing evidence of the cerebellum’s role in emotion processing, the current work aims to update previous meta-analytic findings and compare emotion studies utilizing explicit or implicit allocation of attention to identify common and distinct areas of activation. The present analysis considers studies utilizing broad stimulus classes (primarily visual images) that assess emotion recognition and experience, but excludes studies of social cognition (e.g., theory of mind), emotion regulation, and pain. Based on prior findings [13], it was predicted that explicit emotion tasks would recruit primarily the posterior lateral hemispheres of the cerebellum to support the additional cognitive demands of emotion labelling, while implicit emotion tasks would primarily recruit the vermis to facilitate unconscious affective processing.

## Methods

### Study Selection

**Fig. 1:**
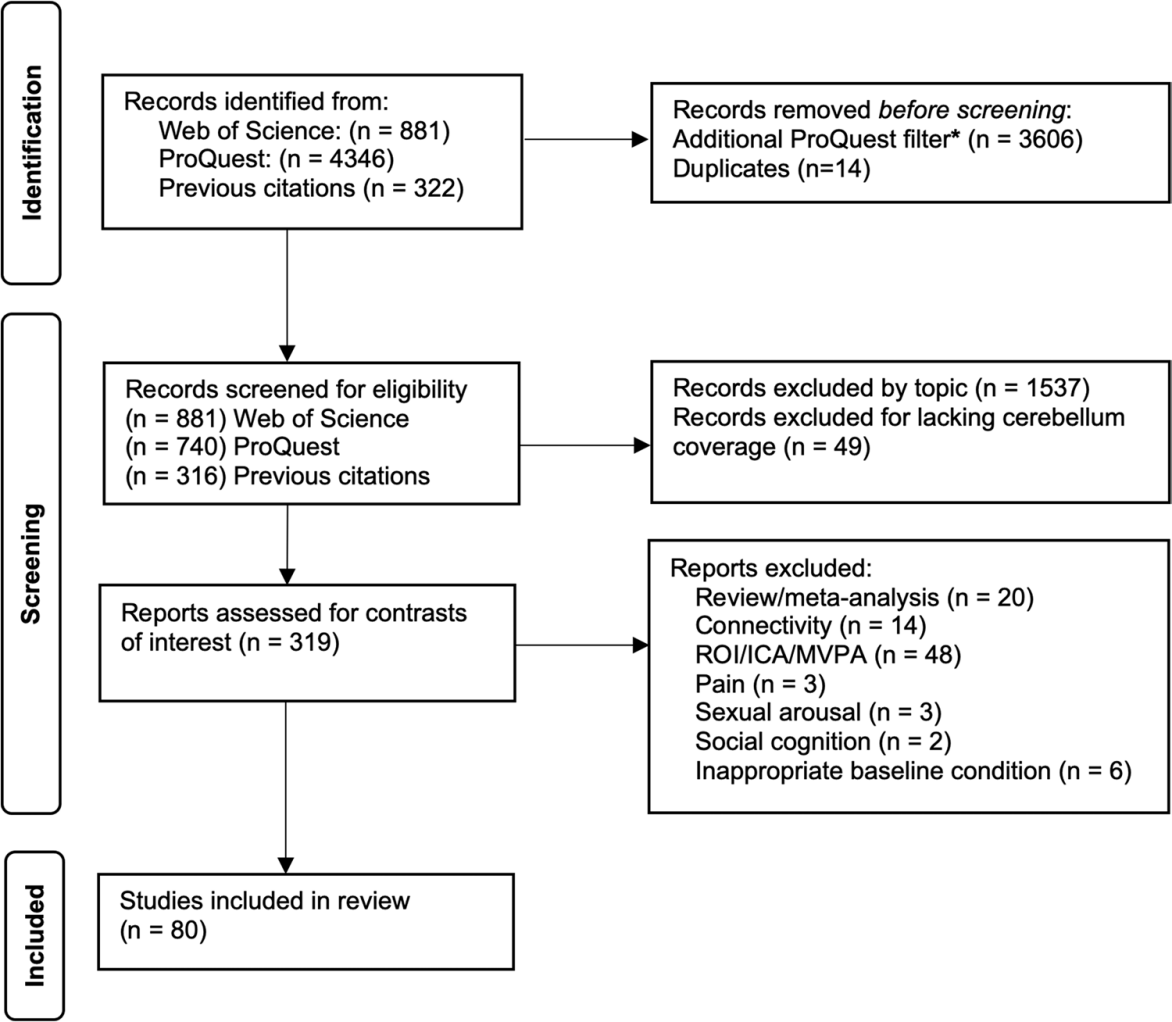
PRISMA flowchart of the literature review and screening process to select human fMRI or PET studies of emotion. Included studies had to report data from healthy adult subjects with whole brain coverage using basic subtraction contrasts with coordinates reported in standard space. *Additionally filtered records automatically for non-relevant topics including “rodents,” “gene expression,” “astronomy,” “drama,” and non-English reports. ROI, region of interest; ICA, independent components analysis; MVPA, multivariate pattern analyses

A literature search was finalized in May 2021 using Web of Science (webofknowledge.com) and ProQuest (proquest.com; includes unpublished studies/theses) with the search terms: fMRI OR PET AND cerebel* AND emotion* OR affect* OR arousal OR motivation* OR apathy OR depression OR anxiety OR mood OR amimia OR aprosodia OR dysprosod* OR "action tendencies" OR "subjective feeling" OR cognition. Additional studies were then extracted from reference lists of prior work to expand study inclusion. This search yielded 881 articles from Web of Science, 740 articles from ProQuest, and 322 articles from previous citations. Details of the screening process are reported in Fig. 1 according to PRISMA guidelines [51]. Individual studies were screened in two steps: (1) two authors independently read the abstracts from each study to exclude any definitively irrelevant or incompatible articles,(2) these authors and one additional author read the full text of each remaining study to determine its adherence to the inclusion criteria: full text available in English, fMRI or PET analysis, emotion-related task, healthy adult subjects (18–60 years old), whole brain coverage that explicitly included the cerebellum (as stated in the methods or evident in the results for any contrast), and basic activation contrasts with coordinates reported in standard space [Montreal Neurological Institute (MNI; [15] or Talairach (Talairach & Tournoux, 1988) atlas].

Studies or contrasts were excluded if they were reviews or meta-analyses, utilized only connectivity, region of interest (ROI), independent/principal components or multivariate pattern analyses (ICA/PCA/MVPA), or if the analysis focused on motor function, social cognition, emotion regulation, pain or sexual arousal. Studies could contribute multiple contrasts/experiments, but redundant contrasts that utilized the same task within a study (such as a conjunction analysis) were excluded [49].

Ultimately, 80 peer-reviewed studies that had been conducted between 1997 and 2021 and included 2761 total participants were selected as meeting the inclusion criteria and addressing the contrasts of interest. Tasks included viewing emotional faces or scenes, smelling pleasant/unpleasant odorants, rating emotional experiences, reading emotional text, and recalling personal emotional memories. Emotions covered by the studies included both positive and negative valence (see Supplementary Material for an exploratory analysis by valence), including specific labels such as happiness, amusement, surprise, sadness, anger, grief, disgust, and fear. The emotional stimuli typically expressed or evoked high arousal and were compared to a neutral or other control condition. Details on the complete list of studies and tasks are provided in Table S2.

Subsequently, study tasks and contrasts were categorized according to the attentional allocation: explicit (attention directed to the type, valence, or intensity of the emotion) or implicit (attention directed to another task or feature of the stimulus), with four contrasts excluded from this secondary analysis for combining both types of attentional tasks. Whole brain coordinates were transformed into MNI space when necessary using GingerALE’s built-in conversion tool. Extracted data is available upon request; this meta-analysis was not pre-registered.

### Analysis

The meta-analysis was performed on the selected studies using an activation likelihood estimation (ALE) approach with the program GingerALE, version 3.0.2 [21, 22, 77]. The program compares peak activation coordinates (foci) across experiments to determine the probability that a given voxel is activated in at least one study. Random effects modeling and methodological modifications to the ALE approach [22, 77] ensure that no single contrast or large sample strongly biases the outcome, and that the results, therefore, best reflect the shared information across studies. The full width half maximum (FWHM) distribution of activation around each point was calculated automatically based on sample size and ranged from 8.5 to 11.4 mm.

In the analysis of whole brain coordinates from all emotion studies, the ALE probabilities were tested for significance against a null distribution using non-parametric testing with 5000 permutations, a voxel level threshold of *p* < 0.01, and cluster level family-wise error correction threshold of *p* < 0.05, a method which accounts for multiple comparisons and the spatial dependence of neuroimaging data [21]. Subsequently, the contrasts were divided into explicit versus implicit attentional categories and separate meta-analyses were conducted to identify any similarities and differences between the two categories using GingerALE’s “Contrast Datasets” function to create conjunction and difference maps. Based on our hypotheses focusing on localization within the cerebellum, whole brain activations (see Supplemental Material Figures S2/S3) from these analyses were masked to and reported only from the cerebellum using a voxel level threshold of *p* < 0.05 with 10,000 permutations and minimum cluster size of 100 mm^3^. A less conservative threshold was implemented for the cerebellum analysis compared to the whole brain analysis based on our a priori hypotheses regarding this region’s contribution to emotion processing and interest in differentiating locations that are most likely to be activated for each emotion category within the cerebellum.

## Results

The classification of study contrasts that met inclusion criteria and addressed the research questions of interest resulted in a total of 139 contrasts from 80 studies on 2761 participants yielding 1404 foci across the whole brain for the combined emotion analysis. The results from the whole brain GingerALE meta-analysis revealed six clusters that were likely to be activated during all emotion studies in the combined analysis: bilateral amygdala, right middle frontal gyrus, bilateral inferior occipital cortex extending to the superior cerebellum, and left parahippocampal gyrus/thalamus (Supplemental Figure S1/Table S1).

Study contrasts then were divided into two categories to further probe the contribution of the cerebellum when attention is directed explicitly toward emotional elements of the stimulus versus when attention is directed elsewhere and the emotional content is processed implicitly. The explicit attention category included 67 contrasts from 36 studies on 1427 participants yielding 661 whole brain foci. The implicit attention category included 68 contrasts from 42 studies on 1277 participants yielding 708 whole brain foci. Four studies were not able to be classified into either the explicit or implicit category and were thus excluded from these analyses, while two studies included separate contrasts for explicit and implicit tasks and thus were included in both categories.

**Fig. 2:**
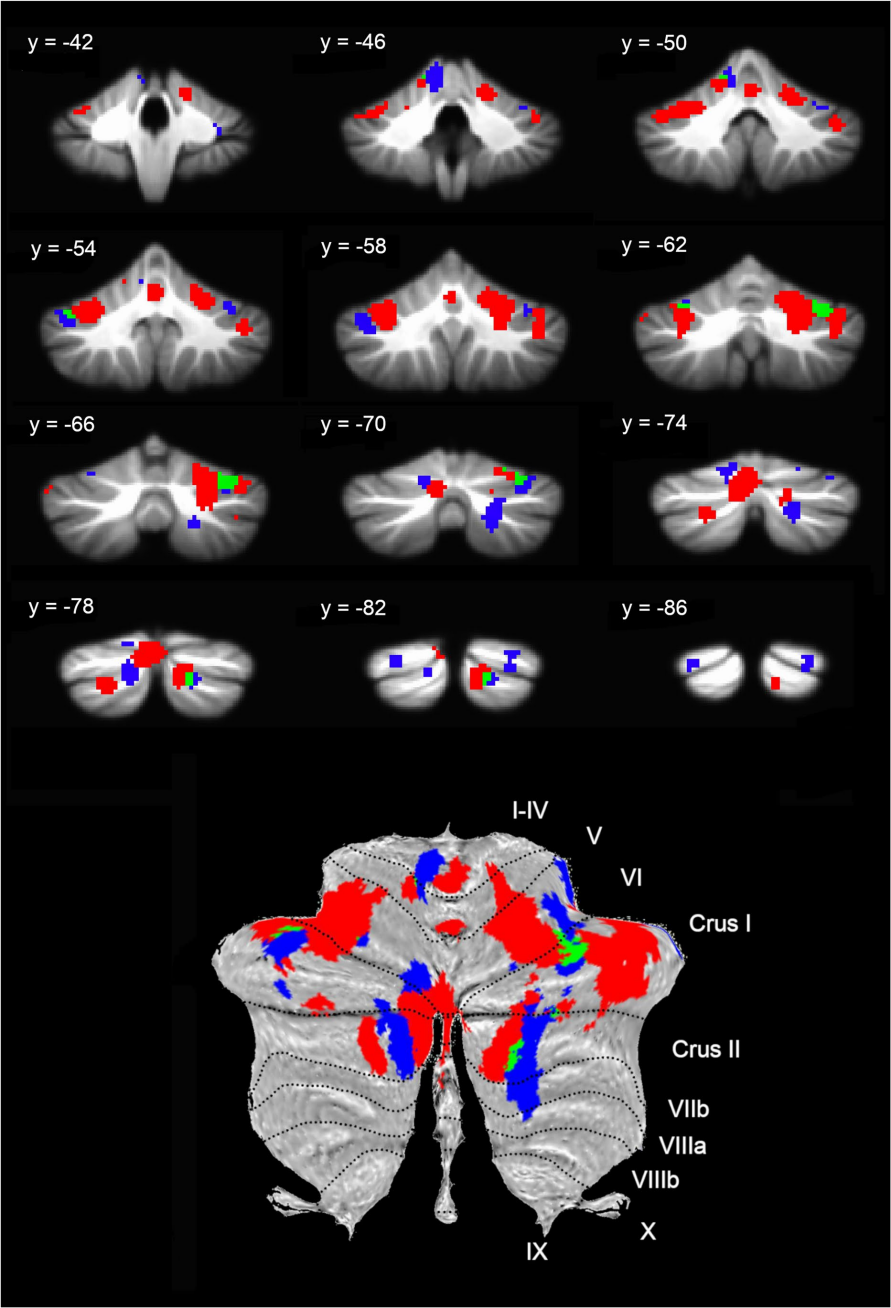
Meta-analysis results for explicit emotion processing (red), implicit emotion processing (blue), and the conjunction of implicit and explicit processing (green) overlaid on (*top*) the SUIT anatomical image slices [18] with MNI y-coordinates and (*bottom*) a flatmap of the SUIT atlas with hemispheric lobule labels; left is shown on the left

Whole brain results for explicit and implicit attention categories are provided in the Supplemental Material (Figures S2/S3/Table S1) and were similar to the results for all studies, but specifically indicated more and larger significant clusters for the implicit versus explicit category, including in bilateral amygdala and right prefrontal cortex. In the cerebellum, for contrasts utilizing explicit attention in emotion tasks, seven clusters were identified that included bilateral lobule VI/Crus I, bilateral Crus I/II, vermis, left Crus II, and left lobules V/VI. For the analysis of implicit attention tasks, ten clusters were identified that included bilateral lobule VI, right Crus II/lobule VIII, left lobules I-IV/V, left lobule VI, bilateral posterior Crus I/II, right anterior lobule VI. (Fig. 2/Table 1). Comparing the peak coordinates with the 7-network parcellation of the cerebellum by Buckner et al. [11] that is available via the online SUIT atlas viewer (https://www.diedrichsenlab.org/imaging/suit.htm), these clusters may be within areas that belong to whole brain functional networks including: executive control, default mode (mentalizing), somatomotor, ventral attention/salience, and limbic networks (Table 1).

**Table 1:** Cluster details for explicit and implicit emotion tasks

Cluster	Size	x	y	z	ALE	Z	Location	Associated functional network
*Explicit attention*								
1	5576	24	− 52	− 20	0.0186	3.47	Right lobule VI/Crus I	Somatomotor
2	2456	− 30	− 58	− 26	0.0181	3.39	Left lateral VI/Crus I	Ventral attention/salience
3	1568	− 6	− 74	− 28	0.0164	3.14	Left Crus II/Vermis/Crus I	Executive control
4	1040	14	− 80	− 38	0.0186	3.47	Right posterior Crus I/Crus II	Default mode
5	504	0	− 54	− 18	0.0128	2.54	Vermis	Limbic
6	448	− 22	− 76	− 40	0.0153	2.95	Left Crus II	Default mode
7	272	− 14	− 50	− 14	0.0121	2.43	Left lobule V/VI	Somatomotor
*Implicit attention*								
8	1080	36	− 66	− 24	0.0171	3.21	Right lobule VI/Crus I	Executive control
9	1064	18	− 80	− 38	0.0133	2.59	Right Crus II/ lobule VIII	Default mode
10	608	− 6	− 46	− 14	0.0119	2.35	Left lobules I-IV/V	Somatomotor
11	464	− 42	− 56	− 30	0.0125	2.45	Left lateral Crus I	Ventral attention/salience
12	384	− 12	− 76	− 20	0.0150	2.87	Left lobule VI	Executive control
13	376	− 10	− 78	− 36	0.0130	2.55	Left posterior Crus II/Crus I	Executive control
14	344	28	− 84	− 26	0.0132	2.56	Right posterior Crus I	Default mode
15	248	− 26	− 84	− 30	0.0123	2.41	Left posterior Crus I	Default mode
16	160	30	− 40	− 36	0.0130	2.54	Right anterior lobule VI	Limbic
17	112	− 30	− 64	− 22	0.0119	2.34	Left lobule VI	Ventral attention/salience
*Explicit and implicit attention*								
18	528	32	− 68	− 28			Right lobule VI/Crus I	Executive control
19	160	16	− 82	− 40			Right Crus II/Crus I	Default mode
20	72	− 40	− 52	− 32			Left lateral Crus I	Ventral attention/ salience
21	40	− 32	− 62	− 24			Left lobule VI	Ventral attention/salience
22	40	− 10	− 48	− 14			Left lobule V	Somatomotor
*Explicit* > *implicit*								
23	1504	28	− 60	− 34			Right lobule VI	Executive control
24	752	− 2	− 78	− 32			Left Vermis, lobule VI	Executive control
25	336	− 32	− 54	− 30			Left lobule VI	Executive control,ventral attention salience
Implicit > explicit								
26	88	− 14	− 78	− 22			Left lobule VI	Executive control

The size (mm^3^) of each cluster, its coordinates in MNI space, maximum ALE value and peak *Z*-statistic (for the overall analysis of each category), and a location description are provided. The associated functional network is based on the 7-network results from Buckner et al. [11], as reported for the peak coordinates in the SUIT atlas viewer

**Fig. 3:**
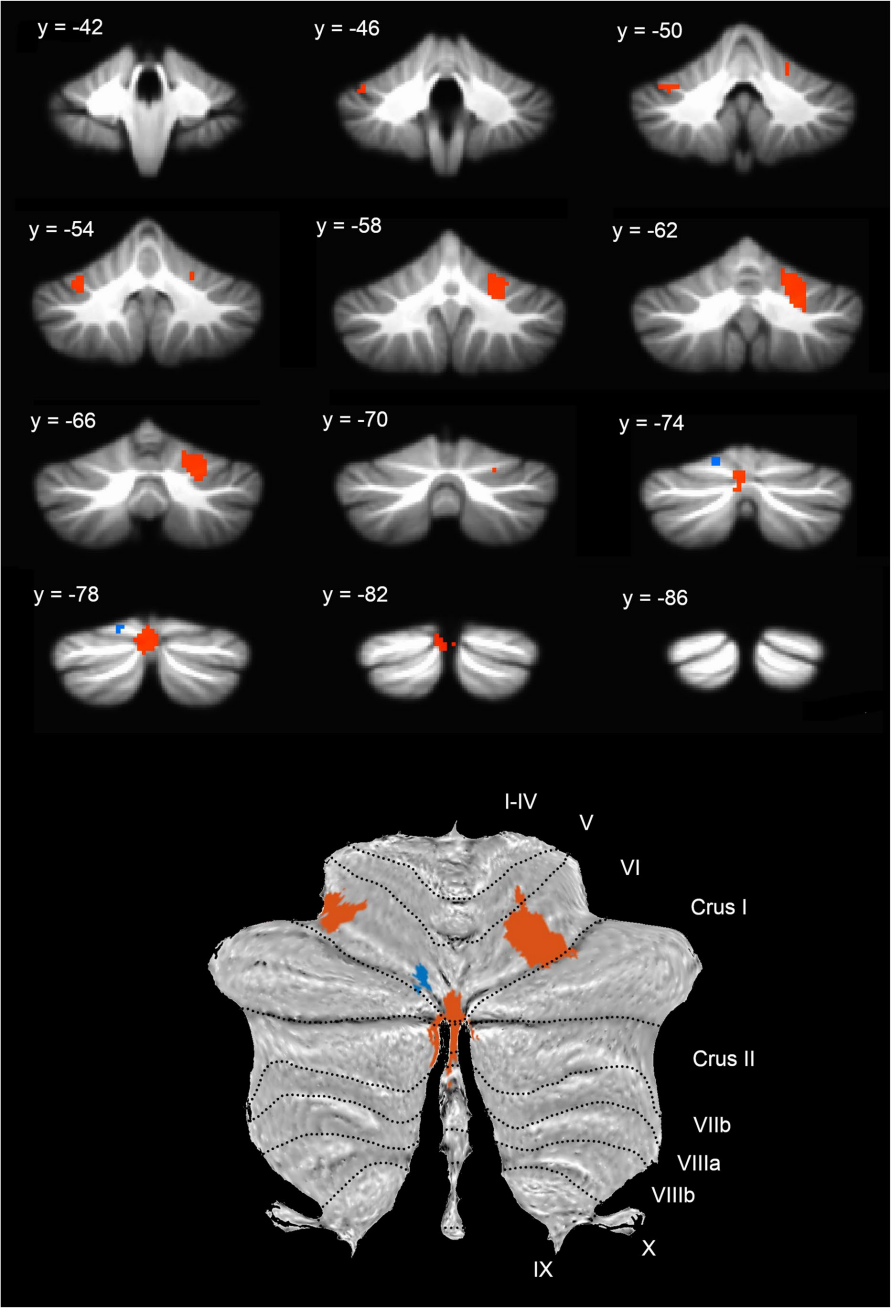
Meta-analysis results directly comparing explicit > implicit (orange) and implicit > explicit (light blue) emotion processing overlaid on (*top*) the SUIT anatomical image with MNI y-coordinates and (*bottom*) a flatmap of the SUIT atlas with hemispheric lobule labels; left is shown on the left

Based on the cerebellar activations for explicit and implicit emotion tasks, the GingerALE “Contrast Datasets” option was used to identify areas of conjunction between the two maps, as well as areas with significantly greater likelihood of activation for each category. The conjunction analysis identified five overlapping clusters including right lobule VI/Crus I, right Crus II/I, left lateral Crus I, and left lobules V/VI. For the explicit category, three clusters with greater likelihood of activation were identified including right lobule VI, left vermis/lobule VI and left lobule VI. For the implicit category, only one small cluster in left lobule VI was more likely to be reported as activated (Fig. 3/Table 1).

As an alternate means of dividing the studies, a contrast analysis was conducted between emotion studies showing/eliciting positive versus negative valence (see Supplemental Material for methods and results). Briefly, several clusters in the cerebellum were significantly likely to be activated by one or both categories including bilateral lobules VI, Crus I, Crus II and the vermis (Figure S4), similar to the attention category analysis. There were three clusters (right Crus I and bilateral lobule VI) more likely to be activated in response to positive valence emotions and one cluster (right lobule VI/Crus I) more likely to be activated by negative valence emotions (Figure S5/Table S3).

## Discussion

In the current meta-analysis of affective functions in the cerebellum, activation foci from 80 neuroimaging studies were combined to identify regions likely to be activated by explicit and/or implicit emotion processing tasks. Whole brain results identified common emotion-related activation in the bilateral amygdala, insula, occipital cortex, superior cerebellum, and right inferior/middle frontal gyrus, consistent with prior reports. The analysis of activation within the cerebellum for explicit emotion tasks identified clusters within the posterior cerebellar hemispheres (bilateral lobule VI/Crus I/II), the vermis, and left lobule V/VI that were likely to be activated across studies, while implicit tasks activated clusters including bilateral lobule VI/Crus I/II, right Crus II/lobule VIII, anterior lobule VI, and lobules I-IV/V. A direct comparison between these categories identified five clusters in the cerebellum for the conjunction of both explicit and implicit tasks, as well as three clusters activated significantly more for explicit emotion tasks compared to implicit tasks, and one cluster activated more for implicit than explicit tasks.

Based on the observed pattern of results, these findings do not support the predicted dissociation of activation in the lateral hemispheric lobules vs. the vermis for emotion tasks requiring explicit attention or implicit attention, respectively. Rather, the present study supports and updates previous meta-analyses indicating distributed activation of the posterior cerebellum during both types of emotion processing in regions that may be associated with neocortical networks supporting cognitive/executive functions, mentalizing, and salience processing. This work highlights the need for researchers to ensure neuroimaging coverage of the cerebellum and to discuss cerebellar activations with respect to the affective and cognitive processes involved in the task (rather than only considering motor function) in order to better characterize how the cerebellum shapes healthy and clinical affective functioning.

### Emotion Processing in the Cerebellum

The results of the meta-analyses for both explicit and implicit emotion studies yielded several clusters in bilateral posterior hemispheric lobule VI and Crus I/II. Additionally, some clusters for each category included portions of the vermis, which in previous task-based and clinical studies has been identified as a crucial region for emotion processing and associative learning (i.e., the “limbic cerebellum”) that is supported by structural connections with limbic subcortical regions and the brainstem [1, 4, 55, 65]. Clinical reports, especially, indicate that the vermis contributes to emotion processing because patients with lesions to this region often display inappropriate emotional responses [66]. There is some evidence, however, that this area of the vermis may be engaged primarily by eye movements and that previous activations of this region in emotional tasks may reflect different visual scanning patterns rather than the affective content itself [36]. The visual nature of many emotional stimuli used in previous studies make this point difficult to disentangle, yet the multimodal nature of the studies included here suggest that eye movements may not be solely driving this activation.

Another cluster located in left lateral Crus I that was identified in the current meta-analysis of both explicit and implicit tasks, also has been associated with emotional tasks in previous studies [48, 71]. The comparison with the Buckner et al. [11] 7-network functional parcellation indicated that this cluster may be associated with the ventral attention/salience network. Such posterior cerebellar regions might be involved in top-down control of affective processing of salient environmental stimuli, with the cerebellum biasing how frontal or parietal cortex responds to sensory input to minimize errors [8]. This proposal is bolstered by clinical evidence from patients with cerebellar lesions of differential electrophysiological activity at frontal and parietal electrodes when viewing angry and fearful faces [2] and increased PET activity in medial prefrontal cortex in response to threatening stimuli [78]. Yet cerebellar patients typically exhibit only mildly impaired emotion recognition capabilities [2, 76], demonstrating that the cerebellum is not directly responsible for generating emotional responses but plays a modulatory role in adapting to the current context and goals [66].

Other clusters in the separate explicit and implicit attention analyses included activation bilaterally in lobules VI, Crus I, and Crus II. In addition to the ventral attention network mentioned above, these clusters were located in regions previously associated with the executive control and default mode neocortical networks [11, 87]. Speculatively, connections with the executive control network may support working memory maintenance of task instructions and endogenous attentional control, while co-activation with the default mode network may indicate self-reflection and mentalizing in order to interpret and identify the evoked emotions [31, 36, 69]. This proposal is further supported by whole brain results indicating activation in prefrontal cortex, especially for implicit attention tasks. Finally, some clusters corresponded to the somatomotor network, which may reflect overt motor responding or facial expressions, or preparation of action tendencies related to the emotional response. Collectively, this pattern of results supports a component process theory of emotion in identifying various affective, cognitive, and motor-related regions that were likely to be activated across the diverse emotion tasks [60].

### Comparison with Previous Meta-analyses

Further insight into the affective functions of the cerebellum can be gained by comparing the current results to two previous meta-analyses that addressed this topic [35, 71]. These studies reported broadly similar results that support a functional topography of the cerebellum that separates sensorimotor and cognitive domains, with the posterior lobe predominantly involved in cognitive and affective processes. In the earliest meta-analysis, Stoodley and Schmahmann [71] compared activations across studies in motor, somatosensory, spatial, language, working memory, executive function, and emotion domains. Their findings included three emotion-related clusters in lateral left Crus I, right lobule VI, and the posterior midline (lobule VII) that roughly correspond to the current analyses, consistent with the fact that the current analysis included most of the same emotion studies, along with newer studies that elicited additional significant clusters in our results.

The second previous meta-analysis [35] included the same study list as Stoodley and Schmahmann [71] for several non-motor functions, which those authors updated. Their results similarly highlighted activations in the bilateral posterior cerebellar lobules including a midline/vermis cluster and a right lobule VI cluster for emotion processing that partially match locations in the current analyses. They reported a left Crus I emotion cluster that was specifically associated with negative emotion processing and is spatially consistent with the results of Stoodley and Schmahmann [71] and the current explicit processing analysis (see also Supplemental Table S3 for current positive vs. negative valence results). The authors also emphasized the overlap of timing-related results with other domains and suggested this function as a critical contribution of the cerebellum to diverse tasks. Specifically, the cerebellum might coordinate the temporal order (i.e., sequence) of different thoughts and actions to improve performance [5, 34, 35, 39, 81, 82] and enforce adequate, ordered synchronization of disparate cortical inputs to achieve a desired output state.

While the current meta-analysis did not include timing or other cognitive domains, by focusing on emotion studies only, the present meta-analysis offers greater detail on the nature of affective processing in the cerebellum by highlighting the similarities and differences between tasks with explicit and implicit attention (as well as positive and negative valence, see Supplemental Material). Whole brain results also indicate regions of co-activation that include the amygdala, insula, occipital cortex, and prefrontal cortex, with more widespread clusters for implicit attention tasks. With the current updated and expanded study list, our work has improved power and greater diversity of tasks and participants to confirm and expand prior findings of emotion-related activation not only in the vermis, but also in bilateral posterior lobules VI, Crus I, and Crus II of the cerebellum.

### Explicit versus Implicit Attention

In consideration of the impact of context on emotional performance, the hypothesis of the current study focused specifically on activation differences between explicit and implicit emotion tasks. Tasks in the former category required explicit attention to the emotion shown or elicited by the stimulus, while tasks in the latter category directed attention to a non-emotional stimulus feature so that emotional information was only processed implicitly. Our direct comparison of studies using explicit versus implicit attention identified clusters that were common to both task types as well as unique to one category or the other. When compared to each other, explicit emotion tasks yielded significantly greater likelihood of activation in three clusters including right lateral lobule VI, the vermis, and left lateral lobule VI, whereas implicit emotion tasks yielded greater activation in only one cluster in left posterior lobule VI. Furthermore, the conjunction analysis identified regions of overlap between the two categories in primarily the posterior and lateral hemispheric lobules.

These findings, therefore, did not support the hypothesis of a dissociation between explicit and implicit attention tasks in which explicit emotion tasks uniquely recruited regions of the posterior cerebellum (that have connections with frontal-parietal and default mode networks) and implicit tasks recruited the vermis (that has connections with the limbic system) [11, 70]. Instead, explicit tasks tended to involve larger portions of the cerebellum including the posterior hemispheres and the vermis, with at least some overlapping regions activated for both explicit and implicit processing. The overlapping recruitment of the posterior lateral hemispheric lobules may indicate that affective functions involving, for example, emotion labelling and automatic physiological changes, are not easily separated during healthy emotion processing and adjacent regions may be activated to a greater or lesser extent according to task demands (cf. [83]. Recruitment of several regions of the cerebellum that may be functionally connected with different neocortical functional networks [11, 36] could help support the various components that contribute to the recognition of emotions and emergence of one’s own emotional experience. Furthermore, differing task demands on attention to emotion across studies impacts the degree to which emotional information is relevant to current goals and, therefore, the extent to which the cerebellum may utilize this information to tune task performance.

Emotional stimuli are likely processed with higher priority than neutral stimuli regardless of the attention instructions that participants receive for a given task, and are automatically appraised for novelty and personal relevance [24, 84], resulting in a physiological response mediated by the vermis that increases when explicitly attended. The need to explicitly report the emotion category or valence could then additionally recruit cognitive and semantic networks for elaborated appraisals of social or motivational significance [30] that rely on the lateral cerebellum to fine-tune and read out the internal model of the emotional experience [1, 56]. Curiously, however, the current whole brain results indicated that implicit tasks recruited broader regions of neocortex, perhaps to override the inherent tendency to process emotional aspects of the stimulus and refocus on the instructed non-emotional task.

## Limitations

One limitation of the findings from the current meta-analysis is that the imaging field of view varied across studies, surely impacting the degree to which the cerebellum was covered during the functional scans and biasing results towards the superior cerebellum. Similarly, individual differences in brain shape and size also make it likely that coverage differed across participants, although the degree of this problem cannot be determined from group maps. Future studies, therefore, should strive to fully include whole brain coverage that is not limited to the neocortex, in order to better estimate the contribution of the inferior cerebellum, which has been shown to contain similar representational maps and connectivity as the superior cerebellum [11].

Another limitation of the current analysis of explicit and implicit studies is that tasks utilizing all individual emotions of both positive and negative valence were included. This choice was made to maximize the number of included studies and examine the effects of explicit and implicit attention regardless of the specific emotion elicited. Nonetheless, there may be differences in how the cerebellum processes individual emotions or valence, perhaps according to the type of behavioral response that is motivated by these categories (see [37]. This proposal was supported by the exploratory meta-analysis of positive versus negative valence contrasts in the current data (see Supplemental Material), which demonstrated differential activation of the cerebellum by valence. Future studies and meta-analyses could investigate in greater detail the effects on cerebellar activation of these and other task design differences that may affect motivation or action tendencies.

## Conclusion

In this meta-analysis, activation foci from prior neuroimaging studies of emotion were combined to identify areas within the cerebellum related to explicit and implicit emotion processing. The analyses of both explicit and implicit categories identified clusters in the bilateral posterior hemispheric lobules, which tended to be more widespread and included the vermis only for explicit tasks. Several of the identified clusters overlapped with regions previously shown to be functionally connected to higher cognitive and limbic networks. Taken together, the results of this meta-analysis of emotion processing suggest that while affective functions are supported by the cerebellum, they do not occur independently of cognitive functions and that tasks using both explicit and implicit attention to emotion recruit numerous cerebellar regions. These findings update previous work investigating affective functions of the cerebellum and highlight the interaction of emotion processing with attention allocation. Ultimately, a better understanding of the functional topography of the cerebellum will lead to improved clinical treatment of patients with cerebellar lesions who have affective or cognitive symptoms.

## Supplementary Information

Below is the link to the electronic supplementary material.

Supplementary file1 (DOC 13.5 MB)
