# Supplementary material for: Explicit and Implicit Emotion Processing in the Cerebellum: A Meta-analysis and Systematic Review
Source: Cerebellum. 2022 Aug 23;22(5):852–64. doi: 10.1007/s12311-022-01459-4 (PMC10485090; doi:10.1007/s12311-022-01459-4)
Supplement: Supplementary file 1 — Supplementary file1 (DOC 13.5 MB) [file 12311_2022_1459_MOESM1_ESM.doc]

Explicit and implicit emotion processing in the cerebellum: a meta-analysis and systematic review

Pierce, Thomasson, Voruz, Selosse, & Péron

Supplemental Material

*The Cerebellum*


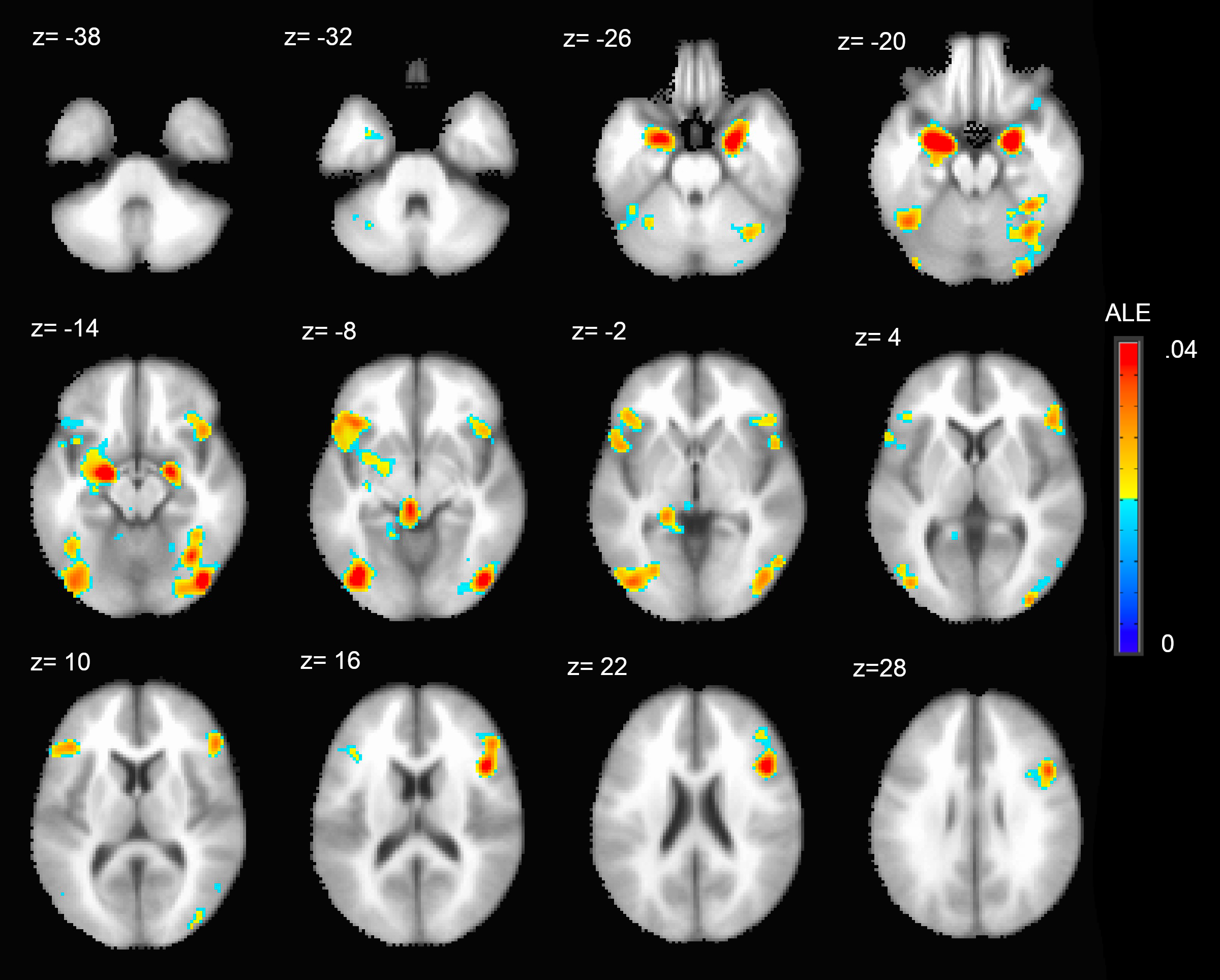


Figure S1. Whole brain results from all 80 emotion studies at a voxel level threshold of *p*<.01 and a cluster level FWE correction threshold of *p*<.05, showing ALE values. This analysis identified six significant clusters (Table S1) including bilateral amygdala, right middle frontal gyrus, bilateral inferior occipital cortex extending to the superior cerebellum, and left parahippocampal gyrus/thalamus.


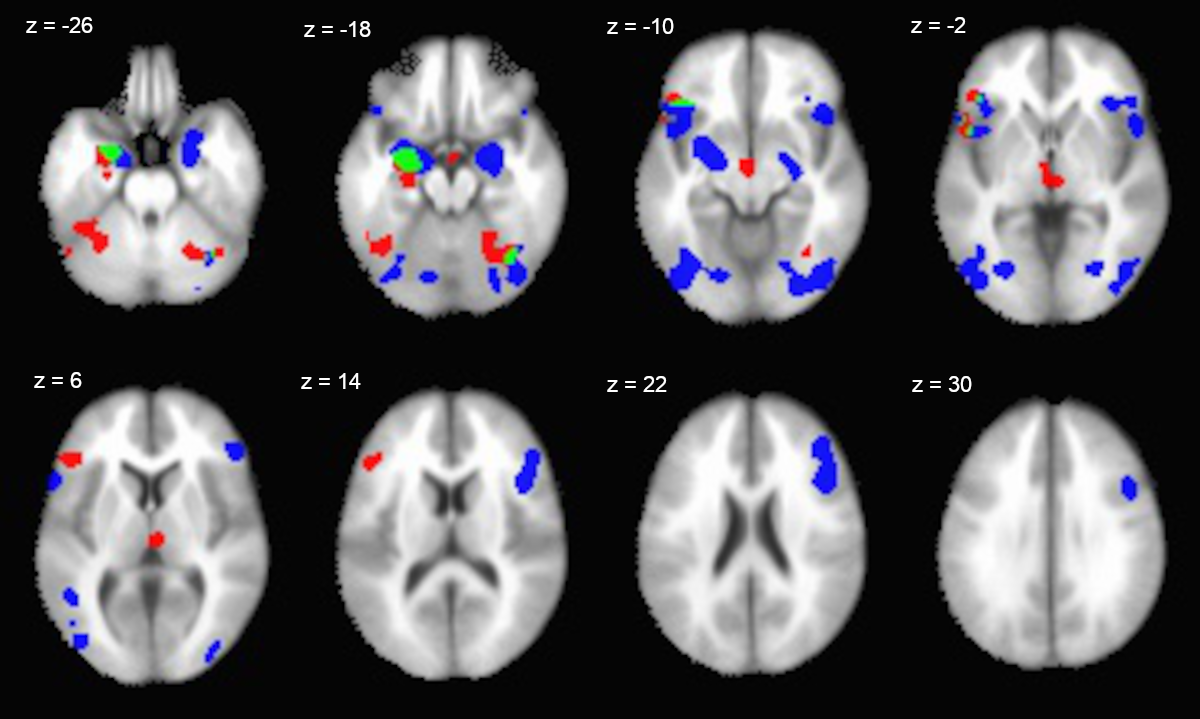


Figure S2. Whole brain results for explicit emotion processing (red), implicit emotion processing (blue), and the conjunction of implicit and explicit processing (green) at a voxel level threshold of *p*<.01 and a cluster level FWE correction threshold of *p*<.05.


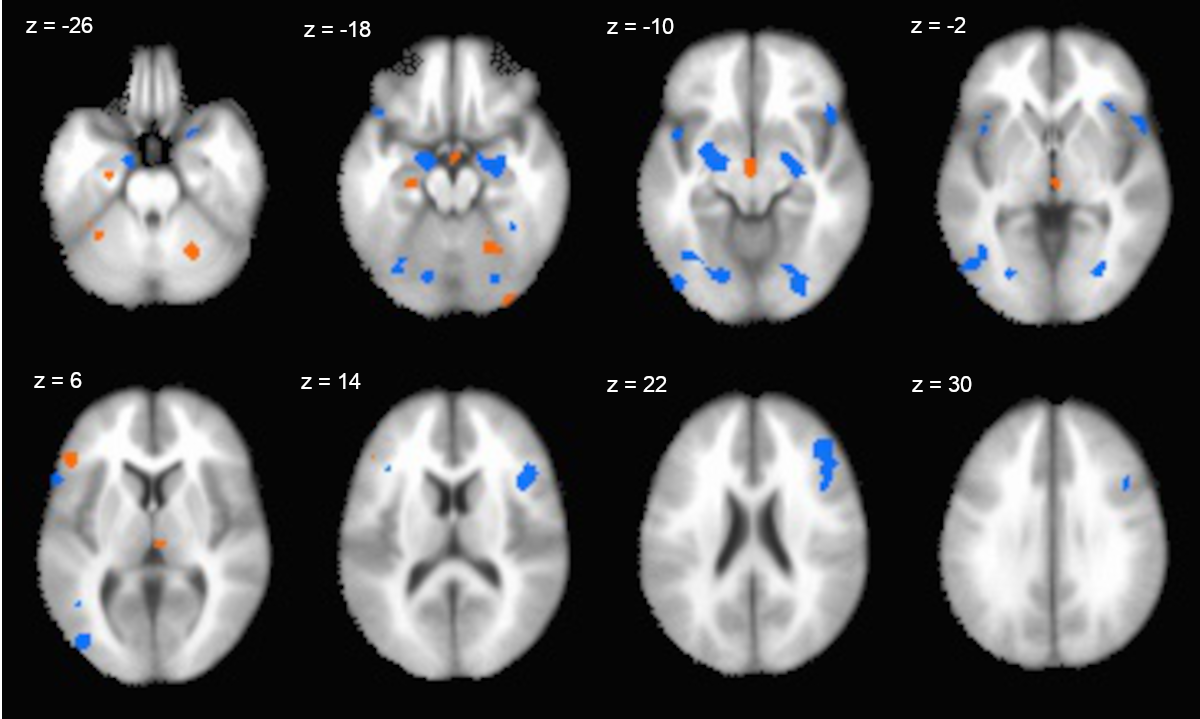


Figure S3. Whole brain results for the analysis directly comparing explicit >implicit (orange) and implicit >explicit (light blue) emotion processing at a voxel level threshold of *p*<.01 and a cluster level FWE correction threshold of *p*<.05.

Table S1. Significant clusters from the whole brain meta-analyses.

| **Cluster** | **Size** | **x** | **y** | **z** | **ALE** | **Z** | **Location** |
| --- | --- | --- | --- | --- | --- | --- | --- |
| *All Studies* | | |  |  |  |  |  |
| 1 | 19352 | -33 | 7 | -11 | 0.0597 | 7.01 | Left amygdala, parahippocampal gyrus, lentiform nucleus, putamen, IFG, insula |
| 2 | 15200 | 38 | -70 | -13 | 0.0514 | 6.24 | Right cerebellum (declive/culmen), fusiform gyrus, MOG, IOG |
| 3 | 12112 | -39 | -70 | -11 | 0.0467 | 5.79 | Left cerebellum (declive/culmen), fusiform gyrus, MOG, IOG |
| 4 | 11968 | 46 | 23 | 10 | 0.0472 | 5.84 | Right IFG, MFG, insula |
| 5 | 5096 | 25 | -4 | -21 | 0.0623 | 7.25 | Right amygdala, parahippocampal gyrus, IFG |
| 6 | 4296 | -10 | -36 | -6 | 0.0391 | 5.01 | Left cerebellum (culmen), parahippocampal gyrus, thalamus |
| *Explicit Attention* | | |  |  |  |  |  |
| 7 | 4392 | 30 | -61 | -20 | 0.0248 | 4.34 | Right cerebellum (declive/culmen), fusiform gyrus |
| 8 | 3944 | -48 | 27 | 2 | 0.0235 | 4.16 | Left IFG, insula, MFG |
| 9 | 3656 | -26 | -8 | -22 | 0.0334 | 5.41 | Left amygdala, hippocampus |
| 10 | 3072 | -39 | -55 | -23 | 0.0190 | 3.53 | Left cerebellum (culmen/declive), fusiform gyrus |
| 11 | 2800 | 1 | -14 | -3 | 0.0216 | 3.90 | Bilateral thalamus, midbrain |
| *Implicit Attention* | | | |  |  |  |  |
| 12 | 11240 | 46 | 23 | 11 | 0.0421 | 6.38 | Right IFG, insula, MFG |
| 13 | 10168 | 37 | -76 | -10 | 0.0313 | 5.13 | Right cerebellum (declive), fusiform gyrus, lingual gyrus, MOG, IOG |
| 14 | 10096 | -37 | -73 | -6 | 0.0322 | 5.25 | Left cerebellum (declive), fusiform gyrus, IOG, lingual gyrus, ITG, MOG |
| 15 | 7688 | -23 | 4 | -18 | 0.0489 | 7.12 | Left parahippocampal gyrus, amygdala, lentiform nucleus, putamen |
| 16 | 5656 | -45 | 19 | -7 | 0.0220 | 3.92 | Left insula, IFG, precentral gyrus |
| 17 | 4992 | 25 | -4 | -19 | 0.0368 | 5.79 | Right parahippocampal gyrus, amygdala, lentiform nucleus, IFG |
| *Explicit and Implicit* | | | |  |  |  |  |
| 18 | 2136 | -26 | -4 | -21 |  |  | Left parahippocampal gyrus, amygdala |
| 19 | 712 | -44 | 28 | -7 |  |  | Left IFG, insula |
| 20 | 608 | 37 | -64 | -20 |  |  | Right cerebellum (declive/culmen) |
| 21 | 144 | -49 | 12 | -1 |  |  | Left insula |
| 22 | 16 | -52 | 19 | -2 |  |  | Left IFG |
| 23 | 8 | -54 | 16 | -2 |  |  | Left IFG |
| *Explicit > Implicit* | | | |  |  |  |  |
| 24 | 1424 | 24 | -60 | -22 |  |  | Right cerebellum (culmen/declive) |
| 25 | 1416 | 0 | -12 | -9 |  |  | Bilateral thalamus, brainstem, hypothalamus |
| 26 | 616 | -49 | 31 | 7 |  |  | Left IFG, MFG |
| 27 | 560 | -26 | -18 | -22 |  |  | Left parahippocampal gyrus, hippocampus |
| 28 | 256 | -34 | -50 | -27 |  |  | Left cerebellum (culmen) |
| 29 | 104 | 34 | -90 | -18 |  |  | Right cerebellum (declive) |
| *Implicit > Explicit* | | | | |  |  |  |
| 30 | 3272 | 45 | 24 | 20 |  |  | Right MFG, SFG |
| 31 | 2760 | -20 | -5 | -15 |  |  | Left lentiform nucleus, parahippocampal gyrus, amygdala, putamen |
| 32 | 2272 | 27 | -77 | -9 |  |  | Right lingual gyrus, fusiform gyrus, cerebellum (declive) |
| 33 | 1920 | 25 | -9 | -14 |  |  | Right parahippocampal gyrus, amygdala, lentiform nucleus |
| 34 | 1872 | -41 | -66 | -6 |  |  | Left cerebellum (declive), ITG, occipital lobe |
| 35 | 1560 | -19 | -76 | -11 |  |  | Left cerebellum (declive), lingual gyrus, fusiform gyrus |
| 36 | 1440 | 48 | 21 | -7 |  |  | Right IFG, insula |
| 37 | 1112 | -43 | -81 | -3 |  |  | Left fusiform gyrus, MOG, IOG, cerebellum (declive) |
| 38 | 520 | -42 | 13 | -7 |  |  | Left insula |
| 39 | 296 | -45 | 23 | -21 |  |  | Left IFG |
| 40 | 280 | -38 | 23 | 17 |  |  | Left IFG |
| 41 | 264 | -58 | 18 | 5 |  |  | Left precentral gyrus, IFG |
| 42 | 200 | 37 | -48 | -20 |  |  | Right cerebellum (culmen), fusiform gyrus |
| 43 | 104 | 25 | 11 | -25 |  |  | Right IFG |

The size (mm^3^) of each cluster, its center of mass coordinates in MNI space, maximum ALE value and peak Z-statistic (for the overall analysis of each category), and a location description are provided. IFG: inferior frontal gyrus; IOG: inferior occipital gyrus; ITG: inferior temporal gyrus; MFG: middle frontal gyrus; MOG: middle occipital gyrus.

Table S2. Study Details

| **Study** | **N (m/f)** | **Age** | **Modality** | **Task** | **Contrast** |
| --- | --- | --- | --- | --- | --- |
| *Explicit Emotion Processing* |  |  |  |  |  |
| Lane et al., 1997 | 0/12 | 18-45 | PET | Rate images | Unpleasant vs. Neutral |
| Reiman et al., 1997 | 0/12 | 23.3 | PET | Mood induction via film clips or recall of personal experience | Film-generated vs. Recall |
| Paradiso et al., 1999 | 7/10 | 31.2 | PET | Rate images | Unpleasant vs. Pleasant |
| Zatorre, Jones-Gotman, & Rouby, 2000 | 6/6 | 23 | PET | Rate odors | Pleasant vs. Baseline |
| Gündel, O'Connor, Littrell, Fort, & Lane, 2003 | 0/8 | NR | 1.5T fMRI | View grief-inducing pictures and words | Word Factor; Picture Factor |
| Kilts, Egan, Gideon, Ely, & Hoffman, 2003 | 9/4 | 24.5 | PET | Rate dynamic and static faces | Dynamic Angry Faces vs. Neutral |
| Lange et al., 2003 | 9/0 | 29 | 1.5T fMRI | Identify emotion while viewing faces | Fear vs. Neutral |
| Markowitsch, Vandekerckhove, Lanfermann, & Russ, 2003 | 6/7 | 30 | PET | Recall emotional memories | Sad vs. Rest |
| Small et al., 2003 | 3/6 | 24 | 1.5T fMRI | Rate taste samples | Unpleasant vs. Pleasant |
| Lee et al., 2004 | 5/5 | 29.5 | 1.5T fMRI | Rate intensity of scenes | Positive vs. Neutral; Negative vs. Neutral |
| Najib, Lorberbaum, Kose, Bohning, & George, 2004 | 0/9 | 25.9 | 1.5T fMRI | Assess ongoing thoughts | Ruminative vs. Neutral |
| Takahashi et al., 2004 | 9/6 | 29.1 | 1.5T fMRI | Categorize positive and negative scenes | Unpleasant vs. Neutral |
| Habel, Klein, Kellermann, Shah, & Schneider, 2005 | 26/0 | 33.4 | 1.5T fMRI | Mood induction via faces | Sadness vs. Control; Happiness vs. Control |
| Hutcherson et al., 2005 | 0/28 | 18-21 | 3T fMRI | Identify emotion of film clips | Amusing vs. Neutral; Sad vs. Neutral |
| Wildgruber et al., 2005 | 5/5 | 22-28 | 1.5T fMRI | Name emotion expressed vocally | Emotion vs. Baseline |
| Bartolo, Benuzzi, Nocetti, Baraldi, & Nichelli, 2006 | 8/13 | 28.1 | 3T fMRI | Rate humor in comics | Funny vs. Neutral |
| Bermpohl et al., 2006 | 8/9 | 21-37 | 3T fMRI | View cued pictures | Emotional Perception vs. Expectance |
| Hofer et al., 2006 | 19/19 | 33.4 | 1.5T fMRI | Mood induction via image viewing | Positive vs. Reference; Negative vs. Reference |
| Hofer et al., 2007 | 19/19 | 33.4 | 1.5T fMRI | Mood induction via words | Positive vs. Reference; Negative vs. Reference |
| Malhi et al., 2007 | 0/10 | 32.4 | 3T fMRI | Identify emotion of faces | Disgust vs. Neutral; Fear vs. Neutral |
| Ogino et al., 2007 | 10/0 | 26.3 | 1.5T fMRI | Mood induction via image viewing | Fear vs. Rest |
| Scheuerecker et al., 2007 | 5/7 | 41.75 | 1.5T fMRI | Match emotional faces | Emotion vs. Identity |
| Payer et al., 2008 | 9/3 | 32.17 | 3T fMRI | Match emotional faces | Emotion vs. Control |
| Colibazzi et al., 2010 | 5/5 | 25.51 | 3T fMRI | Mood induction via sentence reading | Emotional Arousal |
| Fan et al., 2011 | 12/13 | 31.4 | 3T fMRI | Identify emotion of faces | Fear Prime/Fear Target |
| Kohn, Kellermann, Gur, Schneider, & Habel, 2011 | 15/14 | 27 | 3T fMRI | Rate humor of cartoons | Funny vs. Neutral |
| Aupperle et al., 2012 | 10/6 | 23.2 | 3T fMRI | Match emotional faces | Happy vs. Fear; Angry vs. Fear |
| de Greck et al., 2012 | 8/12 | 37 | 1.5T fMRI | Rate ability to empathize with faces | Disgust vs. Control; Anger vs. Control; Joy vs. Control |
| Weisenbach et al., 2012 | 17/21 | 32 | 3T fMRI | Identify emotion of faces | Fear vs. Neutral; Happy vs. Neutral; Angry vs. Neutral; Sad vs. Neutral |
| Fehr, Achtziger, Roth, & Strüber, 2014 | 20/0 | 24.6 | 3T fMRI | View first-person social interaction video | Aggression vs. Neutral; Positive vs. Neutral |
| Ihme et al., 2014a | 26/24 | 23 | 3T fMRI | Identify emotion of faces | Angry vs. Neutral; Fear vs. Neutral; Happy vs. Neutral |
| Ihme et al., 2014b | 25/23 | 24 | 3T fMRI | Identify emotion of faces | Angry vs. Neutral; Fear vs. Neutral; Happy vs. Neutral |
| Osaka, Yaoi, Minamoto, & Osaka, 2014 | 22/11 | 24 | 3T fMRI | View comics | Funny vs. Control |
| Kanat, Heinrichs, Schwarzwald, & Domes, 2015 | 46/0 | 23.64 | 3T fMRI | Recognize emotion of faces | Angry vs. Neutral; Happy vs. Neutral |
| McCloskey et al., 2016 | 12/8 | 32.8 | 3T fMRI | Rate valence of emotional faces | Angry vs. Neutral |
| Coccaro, Keedy, Lee, & Phan, 2021 | 13/13 | 32.0 | 3T fMRI | Rate video clips | Adverse vs. Non-aggressive |
| *Implicit Emotion Processing* |  |  |  |  |  |
| Phillips et al., 1999 | 5 | 30 | 1.5T fMRI | View emotional faces | Angry vs. Control; Fear vs. Control; Disgust vs. Control |
| Phillips et al., 2000 | 7/7 | 31 | 1.5T fMRI | View scenes | Disgust vs. Neutral |
| Simpson et al., 2000 | 9/9 | 24.9 | 1.5T fMRI | View scenes | Negative vs. Neutral Increases |
| Phillips et al., 2001 | 4/2 | 33.8 | 1.5T fMRI | View scenes | Aversive vs. Neutral |
| Williams et al., 2001 | 11/0 | 30 | 1.5T fMRI | View emotional faces | Fear vs. Neutral |
| Iidaka et al., 2002 | 6/6 | 25.1 | 3T fMRI | View emotional faces | Negative vs. Control; Positive vs. Control |
| Abel et al., 2003 | 8/0 | 28.75 | 1.5T fMRI | View emotional faces | Fear vs. Neutral |
| Lange et al., 2003 | 9/0 | 29 | 1.5T fMRI | View emotional faces | Fear vs. Neutral (passive viewing & gender discrimination) |
| Shapira et al., 2003 | 3/5 | 38 | 3T fMRI | View scenes | Disgust vs. Neutral; Threat vs. Neutral |
| Tsukiura, Namiki, Fujii, & Iijima, 2003 | 11/0 | 22.3 | 3T fMRI | Learn face-name pairings | Positive vs. Neutral |
| Wicker et al., 2003 | 14/0 | 20-27 | 3T fMRI | Observe others or smell odorants | Disgust vs. Baseline; Pleasant vs. Baseline |
| Benuzzi et al., 2004 | 7/7 | 21-27 | 1.5T fMRI | View emotional faces | Fear vs. Neutral |
| Hennenlotter et al., 2004 | 5/4 | 35 | 1.5T fMRI | View emotional faces | Disgust vs. Neutral |
| Moran, Wig, Adams, Janata, & Kelley, 2004 | 5/7 | 22-34 | 1.5T fMRI | View TV episodes | Humor Detection; Humor Appreciation |
| Phillips et al., 2004 | 8/0 | 31.9 | 1.5T fMRI | View emotional faces | Overt fear; Overt disgust |
| Schroeder et al., 2004 | 10/10 | 32.5 | 1.5T fMRI | View emotional faces | Surprise vs. Neutral; Disgust vs. Neutral |
| Williams, Das, et al., 2004 | 14/8 | 27.5 | 1.5T fMRI | View emotional faces | Fear vs. Neutral |
| Williams, Brown, et al., 2004 | 15/7 | 20.6 | 1.5T fMRI | View emotional faces | Fear vs. Neutral |
| Hutcherson et al., 2005 | 0/28 | 18-21 | 3T fMRI | View film clips | Amusing vs. Neutral; Sad vs. Neutral |
| Moriguchi et al., 2005 | 12/20 | 29 | 1.5T fMRI | View emotional faces | Fear vs. Neutral |
| Shin et al., 2005 | 13/0 | 49.7 | 1.5T fMRI | View emotional faces | Fear vs. Happy; Happy vs. Fear |
| Deeley et al., 2006 | 9/0 | 27 | 1.5T fMRI | View emotional faces | Fear vs. Neutral; Happy vs. Neutral |
| Sambataro et al., 2006 | 11/13 | 26.8 | 3T fMRI | View emotional faces | Disgust vs. Neutral; Contempt vs. Neutral |
| Deeley et al., 2007 | 9/0 | 27 | 1.5T fMRI | View emotional faces | Fear Intensity; Disgust Intensity; Happy Intensity |
| Hessl et al., 2007 | 13/0 | 39.8 | 1.5T fMRI | View emotional faces | Fear vs. Control; Calm vs. Control |
| Miskowiak et al., 2008 | 7/5 | 23.7 | 1.5T fMRI | View emotional faces | Overt Fear; Overt Happy |
| J. Y. Park et al., 2010 | 7/4 | 23.3 | 1.5T fMRI | Integrate facial and vocal emotion expressions | Angry vs. Neutral |
| Surguladze et al., 2010 | 5/4 | 39.7 | 1.5T fMRI | View emotional faces | Disgust vs. Neutral; Fear vs. Neutral |
| Willems, Clevis, & Hagoort, 2010 | 3/12 | 20.6 | 1.5T fMRI | Read sentences and view images | Sentence Fear vs. Neutral; Combined Fear vs. Neutral |
| Jehna, Langkammer, et al., 2011 | 5/10 | 30.3 | 3T fMRI | View faces and houses | Angry vs. Neutral; Disgust vs. Neutral |
| Jehna, Neuper, et al., 2011 | 9/21 | 36.3 | 3T fMRI | View faces and houses | Angry vs. Neutral; Disgust vs. Neutral |
| Stoodley, Valera, & Schmahmann, 2012 | 9/0 | 25.5 | 3T fMRI | View scenes | Emotional vs. Neutral |
| Mazzola et al., 2013 | 11/12 | 29 | 3T fMRI | View clips of individuals with emotional faces grasping objects | Angry vs. Joyful |
| Pawliczek et al., 2013 | 40/0 | 22.4 | 3T fMRI | Solve anagrams | Unsolvable vs. Solvable |
| Koppe et al., 2015 | 15/15 | 24.2 | 3T fMRI | View emotional faces | Angry vs. Control; Happy vs. Control |
| Castelluccio, Myers, Schuh, & Eigsti, 2016 | 3/5 | 22.68 | 3T fMRI | Listen to sentences | Angry Prosody vs. Neutral |
| Lin et al., 2016 | 6/10 | 22.56 | 3T fMRI | View emotional faces | Angry vs. Happy |
| M.-S. Park, Lee, & Sohn, 2016 | 16/0 | 50.06 | 3T fMRI | View film clips | Angry vs. Neutral |
| Pujol et al., 2018 | 15/15 | 27.9 | 1.5T fMRI | View film clips | Disgust |
| Ziegler et al., 2018 | 13/23 | 24.5 | 3T fMRI | View video clips or read words | Disgust vs. Neutral (faces & words) |
| Sato et al., 2019 | 25/26 | 22.5 | 3T fMRI | Target detection on faces | Dynamic Face vs. Mosaic |
| Zhuang et al., 2021 | 112/115 | 21.62 | 3T fMRI | Go/no-go task with emotional words | Inhibition x Emotion Interaction |
| *Unclassified* |  |  |  |  |  |
| Bruck, Kreifelts, Gossling-Arnold, Wertheimer, & Wildgruber, 2014 | 11/11 | 24.95 | 3T fMRI | Read texts | Emotional Valence |
| Critchley et al., 2000 | 9/0 | 27 | 1.5T fMRI | View emotional faces | Emotion vs. Neutral |
| Narumoto, Okada, Sadato, Fukui, & Yonekura, 2001 | 9/3 | 19-35 | 3T fMRI | Match face identity or emotion | All Tasks vs. Control |
| Seitz et al., 2008 | 7/7 | 28.6 | 1.5T fMRI | Identify emotion of faces | All Tasks vs. Control |

List of studies included in the meta-analysis in each category, with basic descriptions of the study sample and task contrast.

Exploratory Analysis on Emotion Valence in the Cerebellum

**Methods**

To further explore the emotion studies and possible effects of the valence of the emotion being shown or evoked, the 139 contrasts from 80 studies were alternately divided into positive and negative valence categories. As in the primary analyses, study categories were compared using GingerALE’s “Contrast Datasets” function to create conjunction and difference maps. Based on our hypotheses focusing on localization within the cerebellum, whole brain activations from these analyses were masked to and reported only from the cerebellum using a voxel level threshold of *p*<.05 with 10,000 permutations and minimum cluster size of 100 mm^3^.

**Results**

The positive valence category included 37 contrasts from 31 studies on 740 participants yielding 298 whole brain foci. The negative valence category included 91 contrasts from 64 studies on 1618 participants yielding 947 whole brain foci. Nine studies could not be classified into either the positive or negative category and were thus excluded from these analyses, while 24 studies included separate contrasts for positive and negative emotions and thus were included in both categories. The meta-analysis of the positive valence category alone yielded eight clusters in the cerebellum including bilateral lobules VI, Crus I, Crus II, the vermis, and left lobule IX. The analysis of the negative valence category alone yielded nine larger clusters including bilateral lobules V, VI, Crus I, Crus II, vermis, and left lobules I-IV. The conjunction of these two maps yielded seven clusters including bilateral lobules VI and Crus I, and left Crus II (Figure S4/Table S3). The direct comparison of positive > negative valence contrasts resulted in three clusters in right Crus I and bilateral lobule VI, whereas the negative > positive comparison resulted in one significant cluster in right lobule VI/Crus I (Figure S5/Table S3).


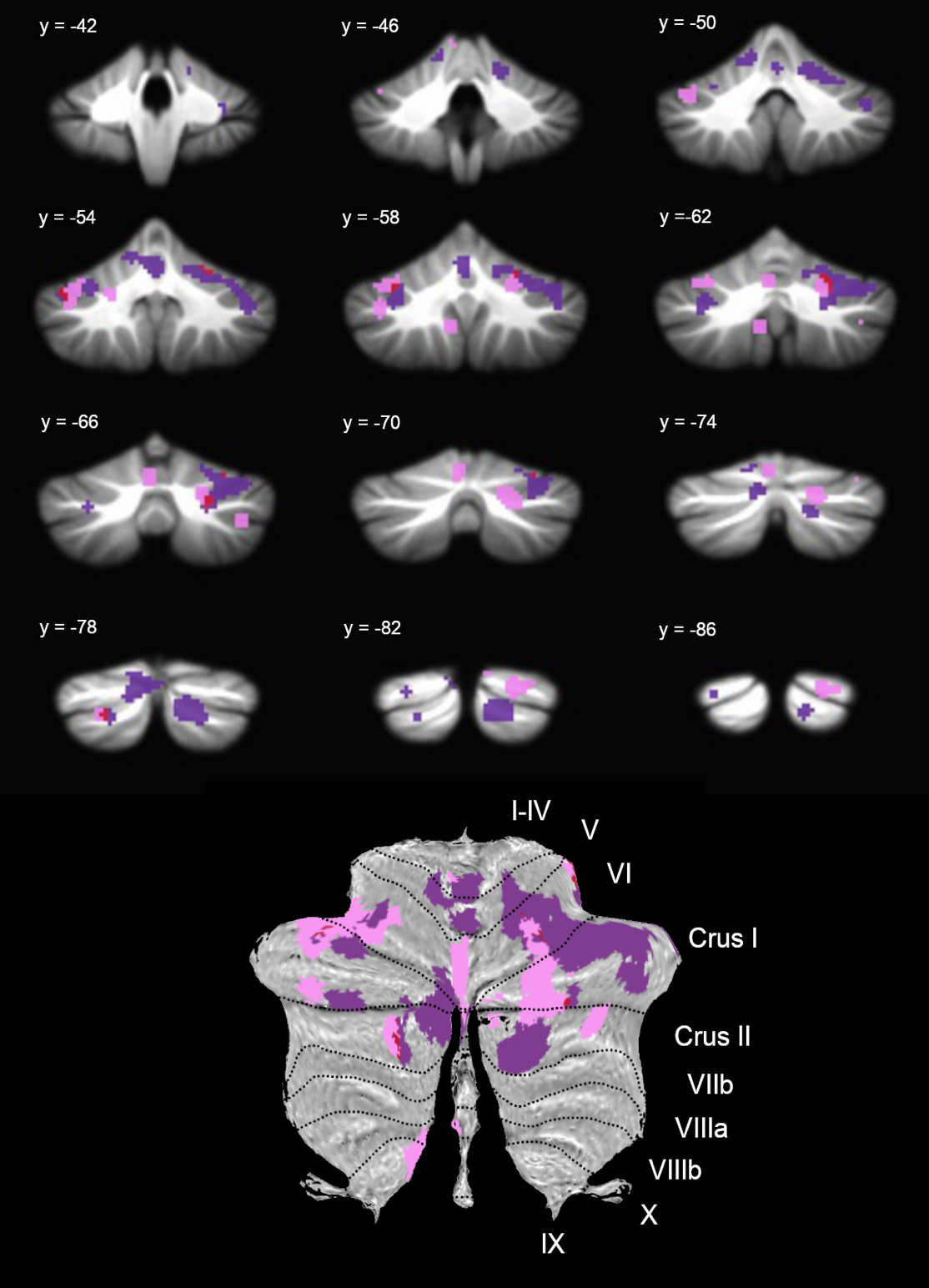


Figure S4. Meta-analysis results for emotion studies with positive valence (pink), negative valence (purple), and the conjunction of positive and negative (magenta) overlaid on (*top*) the SUIT anatomical image with MNI y-coordinates and (*bottom*) a flatmap of the SUIT atlas with hemispheric lobule labels; left is shown on the left.


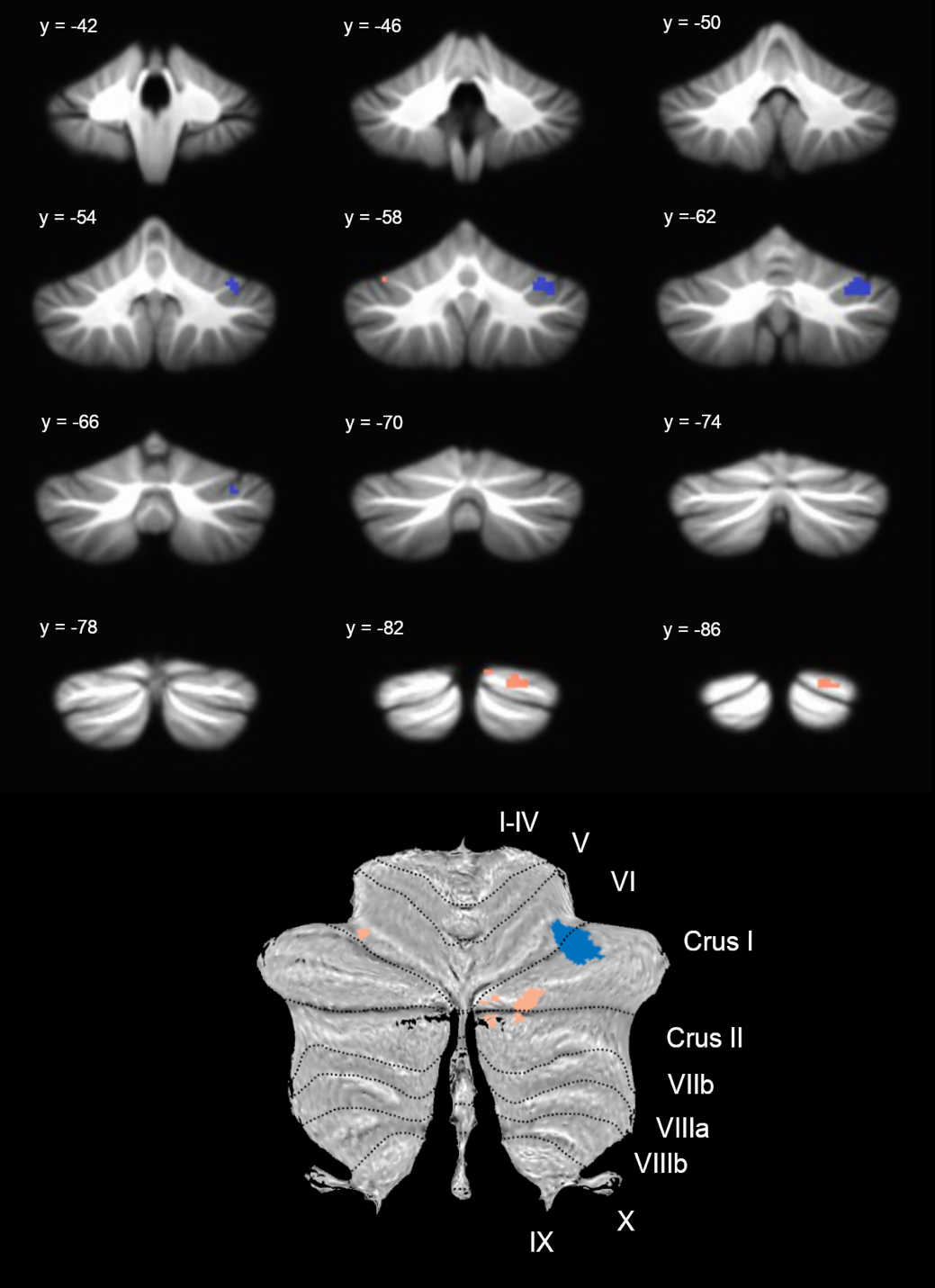


Figure S5. Meta-analysis results directly comparing emotion studies with positive > negative valence (orange) and negative > positive valence (blue) overlaid on (*top*) the SUIT anatomical image with MNI y-coordinates and (*bottom*) a flatmap of the SUIT atlas with hemispheric lobule labels; left is shown on the left.

Table S3. Significant cerebellum clusters from the exploratory meta-analyses of study valence.

| **Cluster** | **Size** | **x** | **y** | **z** | **ALE** | **Z** | **Location** |
| --- | --- | --- | --- | --- | --- | --- | --- |
| *Positive Valence* | | |  |  |  |  |  |
| 1 | 1320 | 22 | -66 | -29 | 0.0096 | 2.68 | Right lobule VI, Crus I |
| 2 | 1136 | -36 | -56 | -29 | 0.0094 | 2.64 | Left lobule VI, Crus I |
| 3 | 656 | 25 | -83 | -28 | 0.0127 | 3.25 | Right Crus I, Crus II |
| 4 | 576 | -2 | -68 | -22 | 0.0079 | 2.33 | Left Vermis, lobule VI, Crus I |
| 5 | 216 | -6 | -60 | -44 | 0.0082 | 2.38 | Left lobule IX, VIIIb, Vermis |
| 6 | 216 | -24 | -78 | -40 | 0.0077 | 2.27 | Left Crus II, Crus I |
| 7 | 200 | 40 | -66 | -42 | 0.0077 | 2.27 | Right Crus II |
| 8 | 184 | -20 | -54 | -30 | 0.0076 | 2.24 | Left lobule VI |
| *Negative Valence* | | | | |  |  |  |
| 9 | 4904 | 30 | -60 | -26 | 0.0271 | 4.29 | Right lobule VI, Crus I, V |
| 10 | 1448 | 16 | -80 | -38 | 0.0269 | 4.13 | Right Crus II, Crus I |
| 11 | 1032 | -4 | -53 | -16 | 0.0154 | 2.64 | Left lobule V, I-IV |
| 12 | 968 | -7 | -77 | -27 | 0.0148 | 2.53 | Left lobule VI, Crus I, Vermis, Crus II |
| 13 | 832 | -30 | -59 | -31 | 0.0171 | 2.88 | Left lobule VI, Crus I |
| 14 | 224 | -21 | -79 | -41 | 0.0133 | 2.28 | Left Crus II |
| 15 | 128 | -27 | -84 | -30 | 0.0123 | 2.10 | Left Crus I, Crus II |
| 16 | 104 | 31 | -41 | -37 | 0.0140 | 2.39 | Right lobule VI |
| 17 | 104 | -42 | -55 | -30 | 0.0115 | 1.96 | Left Crus I |
| *Positive and Negative Valence* | | | | |  |  |  |
| 18 | 152 | 24 | -58 | -22 |  |  | Right lobule VI |
| 19 | 80 | -22 | -78 | -40 |  |  | Left Crus II |
| 20 | 80 | 25 | -67 | -34 |  |  | Right Crus I, lobule VI |
| 21 | 56 | -31 | -59 | -27 |  |  | Left lobule VI |
| 22 | 48 | -41 | -55 | -30 |  |  | Left Crus I |
| 23 | 32 | 33 | -68 | -22 |  |  | Right lobule VI |
| 24 | 24 | 30 | -39 | -37 |  |  | Left lobule VI |
| *Positive > Negative* | | | |  |  |  |  |
| 25 | 328 | 24 | -83 | -27 |  |  | Right Crus I |
| 26 | 40 | 11 | -83 | -24 |  |  | Right Crus I, lobule VI |
| 27 | 16 | -36 | -59 | -24 |  |  | Left lobule VI |
| *Negative > Positive* | | | | |  |  |  |
| 28 | 672 | 37 | -60 | -28 |  |  | Right lobule VI, Crus I |

The size (mm^3^) of each cluster, its center of mass coordinates in MNI space, maximum ALE value and peak Z-statistic (for the overall analysis of each category), and a location description are provided.

**References**

Abel, K. M., Allin, M. P. G., Kucharska-Pietura, K., David, A., Andrew, C., Williams, S., . . . Phillips, M. L. (2003). Ketamine alters neural processing of facial emotion recognition in healthy men: an fMRI study. *Neuroreport, 14*(3), 387-391. Retrieved from https://journals.lww.com/neuroreport/Fulltext/2003/03030/Ketamine_alters_neural_processing_of_facial.18.aspx

Aupperle, R., Tankersley, D., Ravindran, L., Flagan, T., Stein, N., Stein, M., & Paulus, M. (2012). Pregabalin effects on neural response to emotional faces. *Frontiers in Human Neuroscience, 6*. doi:10.3389/fnhum.2012.00042

Bartolo, A., Benuzzi, F., Nocetti, L., Baraldi, P., & Nichelli, P. (2006). Humor comprehension and appreciation: an FMRI study. *J Cogn Neurosci, 18*(11), 1789-1798. doi:10.1162/jocn.2006.18.11.1789

Benuzzi, F., Meletti, S., Zamboni, G., Calandra-Buonaura, G., Serafini, M., Lui, F., . . . Nichelli, P. (2004). Impaired fear processing in right mesial temporal sclerosis: a fMRI study. *Brain Res Bull, 63*(4), 269-281. doi:https://doi.org/10.1016/j.brainresbull.2004.03.005

Bermpohl, F., Pascual-Leone, A., Amedi, A., Merabet, L. B., Fregni, F., Gaab, N., . . . Northoff, G. (2006). Dissociable networks for the expectancy and perception of emotional stimuli in the human brain. *NeuroImage, 30*(2), 588-600. doi:10.1016/j.neuroimage.2005.09.040

Bruck, C., Kreifelts, B., Gossling-Arnold, C., Wertheimer, J., & Wildgruber, D. (2014). 'Inner voices': the cerebral representation of emotional voice cues described in literary texts. *Soc Cogn Affect Neurosci, 9*(11), 1819-1827. doi:10.1093/scan/nst180

Castelluccio, B. C., Myers, E. B., Schuh, J. M., & Eigsti, I. M. (2016). Neural substrates of processing anger in language: contributions of prosody and semantics. *J Psycholinguist Res, 45*(6), 1359-1367. doi:10.1007/s10936-015-9405-z

Coccaro, E. F., Keedy, S., Lee, R., & Phan, K. L. (2021). Neuronal responses to adverse social threat in healthy human subjects. *Journal of Psychiatric Research, 136*, 47-53.

Colibazzi, T., Posner, J., Wang, Z., Gorman, D., Gerber, A., Yu, S., . . . Peterson, B. S. (2010). Neural systems subserving valence and arousal during the experience of induced emotions. *Emotion, 10*(3), 377-389. doi:10.1037/a0018484

Critchley, H., Daly, E., Phillips, M., Brammer, M., Bullmore, E., Williams, S., . . . Murphy, D. (2000). Explicit and implicit neural mechanisms for processing of social information from facial expressions: a functional magnetic resonance imaging study. *Hum Brain Mapp, 9*(2), 93-105. doi:10.1002/(sici)1097-0193(200002)9:2&lt;93::aid-hbm4&gt;3.0.co;2-z

de Greck, M., Scheidt, L., Bolter, A. F., Frommer, J., Ulrich, C., Stockum, E., . . . Northoff, G. (2012). Altered brain activity during emotional empathy in somatoform disorder. *Hum Brain Mapp, 33*(11), 2666-2685. doi:10.1002/hbm.21392

Deeley, Q., Daly, E., Surguladze, S., Tunstall, N., Mezey, G., Beer, D., . . . Murphy, D. G. (2006). Facial emotion processing in criminal psychopathy: Preliminary functional magnetic resonance imaging study. *British Journal of Psychiatry, 189*(6), 533-539. doi:10.1192/bjp.bp.106.021410

Deeley, Q., Daly, E. M., Surguladze, S., Page, L., Toal, F., Robertson, D., . . . Murphy, D. G. M. (2007). An Event Related Functional Magnetic Resonance Imaging Study of Facial Emotion Processing in Asperger Syndrome. *Biol Psychiatry, 62*(3), 207-217. doi:https://doi.org/10.1016/j.biopsych.2006.09.037

Fan, J., Gu, X., Liu, X., Guise, K. G., Park, Y., Martin, L., . . . Hof, P. R. (2011). Involvement of the anterior cingulate and frontoinsular cortices in rapid processing of salient facial emotional information. *NeuroImage, 54*(3), 2539-2546. doi:https://doi.org/10.1016/j.neuroimage.2010.10.007

Fehr, T., Achtziger, A., Roth, G., & Strüber, D. (2014). Neural correlates of the empathic perceptual processing of realistic social interaction scenarios displayed from a first-order perspective. *Brain Research, 1583*, 141-158. doi:https://doi.org/10.1016/j.brainres.2014.04.041

Gündel, H., O'Connor, M. F., Littrell, L., Fort, C., & Lane, R. D. (2003). Functional neuroanatomy of grief: an FMRI study. *Am J Psychiatry, 160*(11), 1946-1953. doi:10.1176/appi.ajp.160.11.1946

Habel, U., Klein, M., Kellermann, T., Shah, N. J., & Schneider, F. (2005). Same or different? Neural correlates of happy and sad mood in healthy males. *NeuroImage, 26*(1), 206-214. doi:https://doi.org/10.1016/j.neuroimage.2005.01.014

Hennenlotter, A., Schroeder, U., Erhard, P., Haslinger, B., Stahl, R., Weindl, A., . . . Ceballos‐Baumann, A. O. (2004). Neural correlates associated with impaired disgust processing in pre‐symptomatic Huntington’s disease. *Brain, 127*(6), 1446-1453. doi:10.1093/brain/awh165

Hessl, D., Rivera, S., Koldewyn, K., Cordeiro, L., Adams, J., Tassone, F., . . . Hagerman, R. J. (2007). Amygdala dysfunction in men with the fragile X premutation. *Brain, 130*(Pt 2), 404-416. doi:10.1093/brain/awl338

Hofer, A., Siedentopf, C. M., Ischebeck, A., Rettenbacher, M. A., Verius, M., Felber, S., & Fleischhacker, W. W. (2006). Gender differences in regional cerebral activity during the perception of emotion: a functional MRI study. *NeuroImage, 32*(2), 854-862. doi:10.1016/j.neuroimage.2006.03.053

Hofer, A., Siedentopf, C. M., Ischebeck, A., Rettenbacher, M. A., Verius, M., Felber, S., & Wolfgang Fleischhacker, W. (2007). Sex differences in brain activation patterns during processing of positively and negatively valenced emotional words. *Psychol Med, 37*(1), 109-119. doi:10.1017/s0033291706008919

Hutcherson, C. A., Goldin, P. R., Ochsner, K. N., Gabrieli, J. D., Barrett, L. F., & Gross, J. J. (2005). Attention and emotion: Does rating emotion alter neural responses to amusing and sad films? *NeuroImage, 27*(3), 656-668. doi:https://doi.org/10.1016/j.neuroimage.2005.04.028

Ihme, K., Sacher, J., Lichev, V., Rosenberg, N., Kugel, H., Rufer, M., . . . Suslow, T. (2014a). Alexithymic features and the labeling of brief emotional facial expressions – An fMRI study. *Neuropsychologia, 64*, 289-299. doi:https://doi.org/10.1016/j.neuropsychologia.2014.09.044

Ihme, K., Sacher, J., Lichev, V., Rosenberg, N., Kugel, H., Rufer, M., . . . Suslow, T. (2014b). Alexithymia and the labeling of facial emotions: response slowing and increased motor and somatosensory processing. *BMC Neuroscience, 15*(1), 40. doi:10.1186/1471-2202-15-40

Iidaka, T., Okada, T., Murata, T., Omori, M., Kosaka, H., Sadato, N., & Yonekura, Y. (2002). Age-related differences in the medial temporal lobe responses to emotional faces as revealed by fMRI. *Hippocampus, 12*(3), 352-362. doi:https://doi.org/10.1002/hipo.1113

Jehna, M., Langkammer, C., Wallner-Blazek, M., Neuper, C., Loitfelder, M., Ropele, S., . . . Enzinger, C. (2011). Cognitively preserved MS patients demonstrate functional differences in processing neutral and emotional faces. *Brain Imaging and Behavior, 5*(4), 241-251. doi:10.1007/s11682-011-9128-1

Jehna, M., Neuper, C., Ischebeck, A., Loitfelder, M., Ropele, S., Langkammer, C., . . . Enzinger, C. (2011). The functional correlates of face perception and recognition of emotional facial expressions as evidenced by fMRI. *Brain Research, 1393*, 73-83. doi:https://doi.org/10.1016/j.brainres.2011.04.007

Kanat, M., Heinrichs, M., Schwarzwald, R., & Domes, G. (2015). Oxytocin Attenuates Neural Reactivity to Masked Threat Cues from the Eyes. *Neuropsychopharmacology, 40*(2), 287-295. doi:10.1038/npp.2014.183

Kilts, C. D., Egan, G., Gideon, D. A., Ely, T. D., & Hoffman, J. M. (2003). Dissociable neural pathways are involved in the recognition of emotion in static and dynamic facial expressions. *NeuroImage, 18*(1), 156-168. doi:10.1006/nimg.2002.1323

Kohn, N., Kellermann, T., Gur, R. C., Schneider, F., & Habel, U. (2011). Gender differences in the neural correlates of humor processing: Implications for different processing modes. *Neuropsychologia, 49*(5), 888-897. doi:https://doi.org/10.1016/j.neuropsychologia.2011.02.010

Koppe, G., Heidel, A., Sammer, G., Bohus, M., Gallhofer, B., Kirsch, P., & Lis, S. (2015). Temporal unpredictability of a stimulus sequence and the processing of neutral and emotional stimuli. *NeuroImage, 120*, 214-224. doi:https://doi.org/10.1016/j.neuroimage.2015.06.081

Lane, R. D., Reiman, E. M., Bradley, M. M., Lang, P. J., Ahern, G. L., Davidson, R. J., & Schwartz, G. E. (1997). Neuroanatomical correlates of pleasant and unpleasant emotion. *Neuropsychologia, 35*(11), 1437-1444. doi:10.1016/s0028-3932(97)00070-5

Lange, K., Williams, L. M., Young, A. W., Bullmore, E. T., Brammer, M. J., Williams, S. C. R., . . . Phillips, M. L. (2003). Task instructions modulate neural responses to fearful facial expressions. *Biol Psychiatry, 53*(3), 226-232. doi:https://doi.org/10.1016/S0006-3223(02)01455-5

Lee, G. P., Meador, K. J., Loring, D. W., Allison, J. D., Brown, W. S., Paul, L. K., . . . Lavin, T. B. (2004). Neural substrates of emotion as revealed by functional magnetic resonance imaging. *Cogn Behav Neurol, 17*(1), 9-17.

Lin, H., Mueller-Bardorff, M., Mothes-Lasch, M., Buff, C., Brinkmann, L., Miltner, W. H. R., & Straube, T. (2016). Effects of Intensity of Facial Expressions on Amygdalar Activation Independently of Valence. *Frontiers in Human Neuroscience, 10*. doi:10.3389/fnhum.2016.00646

Malhi, G. S., Lagopoulos, J., Sachdev, P. S., Ivanovski, B., Shnier, R., & Ketter, T. (2007). Is a lack of disgust something to fear? A functional magnetic resonance imaging facial emotion recognition study in euthymic bipolar disorder patients. *Bipolar Disorders, 9*(4), 345-357. doi:https://doi.org/10.1111/j.1399-5618.2007.00485.x

Markowitsch, H. J., Vandekerckhove, M. M. P., Lanfermann, H., & Russ, M. O. (2003). Engagement of lateral and medial prefrontal areas in the ecphory of sad and happy autobiographical memories. *Cortex, 39*(4), 643-665. doi:https://doi.org/10.1016/S0010-9452(08)70858-X

Mazzola, V., Vuilleumier, P., Latorre, V., Petito, A., Gallese, V., Popolizio, T., . . . Bondolfi, G. (2013). Effects of emotional contexts on cerebello-thalamo-cortical activity during action observation. *PloS one, 8*(9), e75912. doi:10.1371/journal.pone.0075912

McCloskey, M. S., Phan, K. L., Angstadt, M., Fettich, K. C., Keedy, S., & Coccaro, E. F. (2016). Amygdala hyperactivation to angry faces in intermittent explosive disorder. *Journal of Psychiatric Research, 79*, 34-41. doi:https://doi.org/10.1016/j.jpsychires.2016.04.006

Miskowiak, K., Inkster, B., Selvaraj, S., Wise, R., Goodwin, G. M., & Harmer, C. J. (2008). Erythropoietin Improves Mood and Modulates the Cognitive and Neural Processing of Emotion 3 Days Post Administration. *Neuropsychopharmacology, 33*(3), 611-618. doi:10.1038/sj.npp.1301439

Moran, J. M., Wig, G. S., Adams, R. B., Janata, P., & Kelley, W. M. (2004). Neural correlates of humor detection and appreciation. *NeuroImage, 21*(3), 1055-1060. doi:https://doi.org/10.1016/j.neuroimage.2003.10.017

Moriguchi, Y., Ohnishi, T., Kawachi, T., Mori, T., Hirakata, M., Yamada, M., . . . Komaki, G. (2005). Specific brain activation in Japanese and Caucasian people to fearful faces. *Neuroreport, 16*(2), 133-136.

Najib, A., Lorberbaum, J. P., Kose, S., Bohning, D. E., & George, M. S. (2004). Regional brain activity in women grieving a romantic relationship breakup. *Am J Psychiatry, 161*(12), 2245-2256. doi:10.1176/appi.ajp.161.12.2245

Narumoto, J., Okada, T., Sadato, N., Fukui, K., & Yonekura, Y. (2001). Attention to emotion modulates fMRI activity in human right superior temporal sulcus. *Cognitive Brain Research, 12*(2), 225-231. doi:https://doi.org/10.1016/S0926-6410(01)00053-2

Ogino, Y., Nemoto, H., Inui, K., Saito, S., Kakigi, R., & Goto, F. (2007). Inner experience of pain: imagination of pain while viewing images showing painful events forms subjective pain representation in human brain. *Cereb Cortex, 17*(5), 1139-1146. doi:10.1093/cercor/bhl023

Osaka, M., Yaoi, K., Minamoto, T., & Osaka, N. (2014). Serial changes of humor comprehension for four-frame comic Manga: an fMRI study. *Sci Rep, 4*(1), 5828. doi:10.1038/srep05828

Paradiso, S., Johnson, D. L., Andreasen, N. C., O'Leary, D. S., Watkins, G. L., Ponto, L. L., & Hichwa, R. D. (1999). Cerebral blood flow changes associated with attribution of emotional valence to pleasant, unpleasant, and neutral visual stimuli in a PET study of normal subjects. *Am J Psychiatry, 156*(10), 1618-1629. doi:10.1176/ajp.156.10.1618

Park, J. Y., Gu, B. M., Kang, D. H., Shin, Y. W., Choi, C. H., Lee, J. M., & Kwon, J. S. (2010). Integration of cross-modal emotional information in the human brain: an fMRI study. *Cortex, 46*(2), 161-169. doi:10.1016/j.cortex.2008.06.008

Park, M.-S., Lee, B. H., & Sohn, J.-H. (2016). Neural substrates involved in anger induced by audio-visual film clips among patients with alcohol dependency. *Journal of Physiological Anthropology, 36*(1), 5. doi:10.1186/s40101-016-0102-x

Pawliczek, C. M., Derntl, B., Kellermann, T., Gur, R. C., Schneider, F., & Habel, U. (2013). Anger under Control: Neural Correlates of Frustration as a Function of Trait Aggression. *PloS one, 8*(10), e78503. doi:10.1371/journal.pone.0078503

Payer, D. E., Lieberman, M. D., Monterosso, J. R., Xu, J., Fong, T. W., & London, E. D. (2008). Differences in cortical activity between methamphetamine-dependent and healthy individuals performing a facial affect matching task. *Drug and Alcohol Dependence, 93*(1), 93-102. doi:https://doi.org/10.1016/j.drugalcdep.2007.09.009

Phillips, M. L., Marks, I. M., Senior, C., Lythgoe, D., O'Dwyer, A. M., Meehan, O., . . . McGuire, P. K. (2000). A differential neural response in obsessive–compulsive disorder patients with washing compared with checking symptoms to disgust. *Psychol Med, 30*(5), 1037-1050. doi:10.1017/S0033291799002652

Phillips, M. L., Medford, N., Senior, C., Bullmore, E. T., Suckling, J., Brammer, M. J., . . . David, A. S. (2001). Depersonalization disorder: thinking without feeling. *Psychiatry Research: Neuroimaging, 108*(3), 145-160. doi:https://doi.org/10.1016/S0925-4927(01)00119-6

Phillips, M. L., Williams, L., Senior, C., Bullmore, E. T., Brammer, M. J., Andrew, C., . . . David, A. S. (1999). A differential neural response to threatening and non-threatening negative facial expressions in paranoid and non-paranoid schizophrenics. *Psychiatry Research: Neuroimaging, 92*(1), 11-31. doi:https://doi.org/10.1016/S0925-4927(99)00031-1

Phillips, M. L., Williams, L. M., Heining, M., Herba, C. M., Russell, T., Andrew, C., . . . Gray, J. A. (2004). Differential neural responses to overt and covert presentations of facial expressions of fear and disgust. *NeuroImage, 21*(4), 1484-1496. doi:https://doi.org/10.1016/j.neuroimage.2003.12.013

Pujol, J., Blanco‐Hinojo, L., Coronas, R., Esteba‐Castillo, S., Rigla, M., Martínez‐Vilavella, G., . . . Caixàs, A. (2018). Mapping the sequence of brain events in response to disgusting food. *Hum Brain Mapp, 39*(1), 369-380.

Reiman, E. M., Lane, R. D., Ahern, G. L., Schwartz, G. E., Davidson, R. J., Friston, K. J., . . . Chen, K. (1997). Neuroanatomical correlates of externally and internally generated human emotion. *Am J Psychiatry, 154*(7), 918-925. doi:10.1176/ajp.154.7.918

Sambataro, F., Dimalta, S., Di Giorgio, A., Taurisano, P., Blasi, G., Scarabino, T., . . . Bertolino, A. (2006). Preferential responses in amygdala and insula during presentation of facial contempt and disgust. *European Journal of Neuroscience, 24*(8), 2355-2362.

Sato, W., Kochiyama, T., Uono, S., Sawada, R., Kubota, Y., Yoshimura, S., & Toichi, M. (2019). Widespread and lateralized social brain activity for processing dynamic facial expressions. *Hum Brain Mapp, 40*(13), 3753-3768.

Scheuerecker, J., Frodl, T., Koutsouleris, N., Zetzsche, T., Wiesmann, M., Kleemann, A. M., . . . Meisenzahl, E. M. (2007). Cerebral differences in explicit and implicit emotional processing--an fMRI study. *Neuropsychobiology, 56*(1), 32-39. doi:10.1159/000110726

Schroeder, U., Hennenlotter, A., Erhard, P., Haslinger, B., Stahl, R., Lange, K. W., & Ceballos-Baumann, A. O. (2004). Functional neuroanatomy of perceiving surprised faces. *Hum Brain Mapp, 23*(4), 181-187. doi:https://doi.org/10.1002/hbm.20057

Seitz, R. J., Schäfer, R., Scherfeld, D., Friederichs, S., Popp, K., Wittsack, H. J., . . . Franz, M. (2008). Valuating other people’s emotional face expression: a combined functional magnetic resonance imaging and electroencephalography study. *Neuroscience, 152*(3), 713-722. doi:https://doi.org/10.1016/j.neuroscience.2007.10.066

Shapira, N. A., Liu, Y., He, A. G., Bradley, M. M., Lessig, M. C., James, G. A., . . . Goodman, W. K. (2003). Brain activation by disgust-inducing pictures in obsessive-compulsive disorder. *Biol Psychiatry, 54*(7), 751-756. doi:https://doi.org/10.1016/S0006-3223(03)00003-9

Shin, L. M., Wright, C. I., Cannistraro, P. A., Wedig, M. M., McMullin, K., Martis, B., . . . Krangel, T. S. (2005). A functional magnetic resonance imaging study of amygdala and medial prefrontal cortex responses to overtly presented fearful faces in posttraumatic stress disorder. *Arch Gen Psychiatry, 62*(3), 273-281.

Simpson, J. R., Ongür, D., Akbudak, E., Conturo, T. E., Ollinger, J. M., Snyder, A. Z., . . . Raichle, M. E. (2000). The emotional modulation of cognitive processing: an fMRI study. *J Cogn Neurosci, 12 Suppl 2*, 157-170. doi:10.1162/089892900564019

Small, D. M., Gregory, M. D., Mak, Y. E., Gitelman, D., Mesulam, M. M., & Parrish, T. (2003). Dissociation of neural representation of intensity and affective valuation in human gustation. *Neuron, 39*(4), 701-711. doi:10.1016/s0896-6273(03)00467-7

Stoodley, C. J., Valera, E. M., & Schmahmann, J. D. (2012). Functional topography of the cerebellum for motor and cognitive tasks: an fMRI study. *NeuroImage, 59*(2), 1560-1570. doi:10.1016/j.neuroimage.2011.08.065

Surguladze, S. A., El-Hage, W., Dalgleish, T., Radua, J., Gohier, B., & Phillips, M. L. (2010). Depression is associated with increased sensitivity to signals of disgust: A functional magnetic resonance imaging study. *Journal of Psychiatric Research, 44*(14), 894-902. doi:https://doi.org/10.1016/j.jpsychires.2010.02.010

Takahashi, H., Koeda, M., Oda, K., Matsuda, T., Matsushima, E., Matsuura, M., . . . Okubo, Y. (2004). An fMRI study of differential neural response to affective pictures in schizophrenia. *NeuroImage, 22*(3), 1247-1254. doi:https://doi.org/10.1016/j.neuroimage.2004.03.028

Tsukiura, T., Namiki, M., Fujii, T., & Iijima, T. (2003). Time-dependent neural activations related to recognition of people's names in emotional and neutral face-name associative learning: an fMRI study. *NeuroImage, 20*(2), 784-794. doi:10.1016/s1053-8119(03)00378-1

Weisenbach, S. L., Rapport, L. J., Briceno, E. M., Haase, B. D., Vederman, A. C., Bieliauskas, L. A., . . . Langenecker, S. A. (2012). Reduced emotion processing efficiency in healthy males relative to females. *Social cognitive and affective neuroscience, 9*(3), 316-325. doi:10.1093/scan/nss137

Wicker, B., Keysers, C., Plailly, J., Royet, J.-P., Gallese, V., & Rizzolatti, G. (2003). Both of Us Disgusted in My Insula: The Common Neural Basis of Seeing and Feeling Disgust. *Neuron, 40*(3), 655-664. doi:https://doi.org/10.1016/S0896-6273(03)00679-2

Wildgruber, D., Riecker, A., Hertrich, I., Erb, M., Grodd, W., Ethofer, T., & Ackermann, H. (2005). Identification of emotional intonation evaluated by fMRI. *NeuroImage, 24*(4), 1233-1241. doi:10.1016/j.neuroimage.2004.10.034

Willems, R. M., Clevis, K., & Hagoort, P. (2010). Add a picture for suspense: neural correlates of the interaction between language and visual information in the perception of fear. *Social cognitive and affective neuroscience, 6*(4), 404-416. doi:10.1093/scan/nsq050

Williams, L. M., Brown, K. J., Das, P., Boucsein, W., Sokolov, E. N., Brammer, M. J., . . . Gordon, E. (2004). The dynamics of cortico-amygdala and autonomic activity over the experimental time course of fear perception. *Cognitive Brain Research, 21*(1), 114-123. doi:https://doi.org/10.1016/j.cogbrainres.2004.06.005

Williams, L. M., Das, P., Harris, A. W., Liddell, B. B., Brammer, M. J., Olivieri, G., . . . Peduto, A. (2004). Dysregulation of arousal and amygdala-prefrontal systems in paranoid schizophrenia. *American Journal of Psychiatry, 161*(3), 480-489.

Williams, L. M., Phillips, M. L., Brammer, M. J., Skerrett, D., Lagopoulos, J., Rennie, C., . . . Gordon, E. (2001). Arousal Dissociates Amygdala and Hippocampal Fear Responses: Evidence from Simultaneous fMRI and Skin Conductance Recording. *NeuroImage, 14*(5), 1070-1079. doi:https://doi.org/10.1006/nimg.2001.0904

Zatorre, R. J., Jones-Gotman, M., & Rouby, C. (2000). Neural mechanisms involved in odor pleasantness and intensity judgments. *Neuroreport, 11*(12), 2711-2716. doi:10.1097/00001756-200008210-00021

Zhuang, Q., Xu, L., Zhou, F., Yao, S., Zheng, X., Zhou, X., . . . Li, K. (2021). Segregating domain-general from emotional context-specific inhibitory control systems-ventral striatum and orbitofrontal cortex serve as emotion-cognition integration hubs. *NeuroImage*, 118269.

Ziegler, J. C., Montant, M., Briesemeister, B. B., Brink, T. T., Wicker, B., Ponz, A., . . . Braun, M. (2018). Do Words Stink? Neural Reuse as a Principle for Understanding Emotions in Reading. *J Cogn Neurosci, 30*(7), 1023-1032. doi:10.1162/jocn_a_01268
